# Blue Light Emitting Polyphenylene Dendrimers with Bipolar Charge Transport Moieties

**DOI:** 10.3390/molecules21101400

**Published:** 2016-10-20

**Authors:** Guang Zhang, Manuel Auer-Berger, Dominik W. Gehrig, Paul W. M. Blom, Martin Baumgarten, Dieter Schollmeyer, E. J. W. List-Kratochvil, Klaus Müllen

**Affiliations:** 1 Max Planck Institute for Polymer Research, Ackermannweg 10, Mainz D-55128, Germany; zhangg@mpip-mainz.mpg.de (G.Z.); Dominik.Gehrig@web.de (D.W.G.); blom@mpip-mainz.mpg.de (P.W.M.B.); 2 Joanneum Research Materials, Institut für Oberflächentechnologien und Photonik, Franz-Pichler-Straße 30, Weiz 8160, Austria; Manuel.Auer-Berger@joanneum.at; 3 Institute für Organische Chemie, Johannes Gutenberg-Universität, Mainz D-55128, Germany; scholli@uni-mainz.de; 4 Institut für Physik, Institut für Chemie, Humboldt-Universität zu Berlin, IRIS Adlershof, Unter den Linden 6, Berlin 10099, Germany

**Keywords:** blue light emitter, bipolar charge transport, polyphenylene dendrimer, surface to core energy transfer

## Abstract

Two light-emitting polyphenylene dendrimers with both hole and electron transporting moieties were synthesized and characterized. Both molecules exhibited pure blue emission solely from the pyrene core and efficient surface-to-core energy transfers when characterized in a nonpolar environment. In particular, the carbazole- and oxadiazole-functionalized dendrimer (**D1**) manifested a pure blue emission from the pyrene core without showing intramolecular charge transfer (ICT) in environments with increasing polarity. On the other hand, the triphenylamine- and oxadiazole-functionalized one (**D2**) displayed notable ICT with dual emission from both the core and an ICT state in highly polar solvents. **D1**, in a three-layer organic light emitting diode (OLED) by solution processing gave a pure blue emission with Commission Internationale de l’Éclairage 1931 CIE *xy* = (0.16, 0.12), a peak current efficiency of 0.21 cd/A and a peak luminance of 2700 cd/m^2^. This represents the first reported pure blue dendrimer emitter with bipolar charge transport and surface-to-core energy transfer in OLEDs.

## 1. Introduction

OLEDs have manifested a broad range of applications in flat-panel displays and solid-state lighting [1,2,3]. However, blue OLEDs still suffer from much shorter lifetimes than green and red ones [4,5], even though pure blue light emitters have demonstrated over 20% EQE for phosphorescent materials [6,7] and nearly 20% for thermally activated delayed fluorescence (TADF) molecules [8]. Dendrimer-based OLEDs have been investigated in the last two decades, due to their numerous advantages over small molecules and polymers, i.e., cost-effective solution processability, high performance reproducibility thanks to their well-defined structures, in contrast to polymers, and precise functionalization of dendrimers at multiple positions [9,10,11,12,13]. Dendrimers for OLEDs generally include two types: one designed for better charge transport (conjugated scaffold) [14,15,16,17,18,19] and one for surface-to-core energy transfers (non-conjugated scaffold) [20,21,22,23,24].

**Figure 1:**
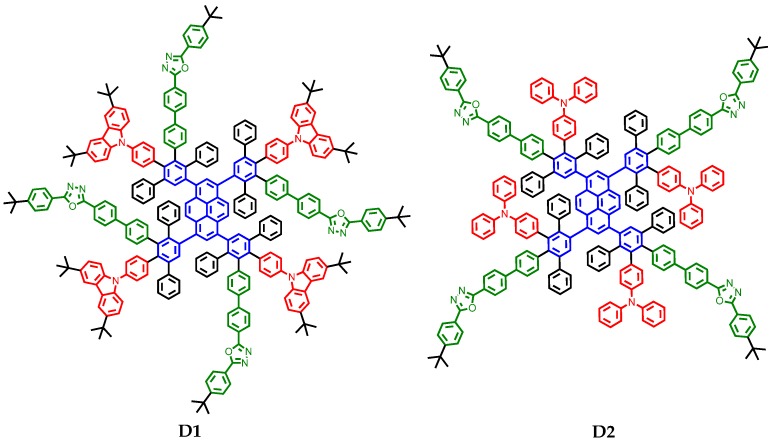
Molecular structures of donor-acceptor dendrimers **D1** and **D2**.

For this purpose, shape-persistent polyphenylene dendrimers enable generation-by-generation (core-shell-surface) functionalization and OLEDs utilizing efficient surface-to-core energy transfers of pyrene-cored polyphenylene dendrimers have been reported [22,23,24]. For example, **PYNPA** (a pyrene-cored dendrimer with 1-naphthyl-*N*,*N*-diphenylamine moieties in the periphery; see Supplementary Materials) achieved better device performance (e.g., 0.24 cd/A and 715 cd/m^2^ in a single-layer device) than other dendrimers due to the favorable HOMO/LUMO levels of the 1-naphthyl-*N*,*N*-diphenylamine moieties for both hole and electron injections in a device [23,24]. Therefore, it is reasonable to expect that a dendrimer with PCs of both hole and electron (bipolar) transport properties could further facilitate charge injection and transport and improve device performance. A good way to produce a bipolar transporting material is to introduce both donor and acceptor components in a single molecule, a technique which has been frequently utilized to make bipolar small-molecular emitters [25,26] and host materials [27]. However, the number of donor-acceptor dendrimers with surface-to-core energy transfer is rather limited [28,29]. To the best of our knowledge, no such blue-light-emitting dendrimers have been reported yet. The difficulty to design this kind of material is the frequently encountered donor-acceptor charge transfer state (between the peripheral chromophores), which causes strong bathochromic emission [30,31,32,33], sometimes notable fluorescence quenching [32,33] and, less commonly, dual emission (localized and ICT emission) [34,35], and this bathochromic emission from the donor-acceptor surface of a dendrimer could easily disable surface-to-core Förster resonance energy transfer due to the high energy gap of a blue-light-emitting core. Bryce et al. reported that small molecules with carbazole and oxadiazole moieties exhibited deep blue emission in thin films even though notable donor-acceptor charge transfer occurred in polar solvents [36]. We therefore decided to use the compounds 3,6-di-*tert*-butyl-9-phenylcarbazole (**CAB**) and 2-(4-*tert*-butylphenyl)-5-(4-biphenyl)-1,3,4-oxadiazole (**PBD**) as the peripheral chromophores of our target dendrimer, i.e., **D1** (Figure 1). In addition, a **TPA**- and **PBD**-functionalized dendrimer **D2** was prepared for comparison. The emission of **CAB** [37], **PBD** [38] and **TPA** [39] show good overlaps with the absorption of the core, i.e., 1,3,6,8-tetraphenylpyrene [40,41], for efficient surface-to-core energy transfers.

To summarize, in this contribution a new method to make polyphenylene dendrimers with donor-acceptor periphery is introduced. Their synthesis, photophysical and electrochemical characterizations together with the device performances are described and discussed.

## 2. Results and Discussion

### 2.1. Synthesis and Structural Characterization

**Scheme 1:**
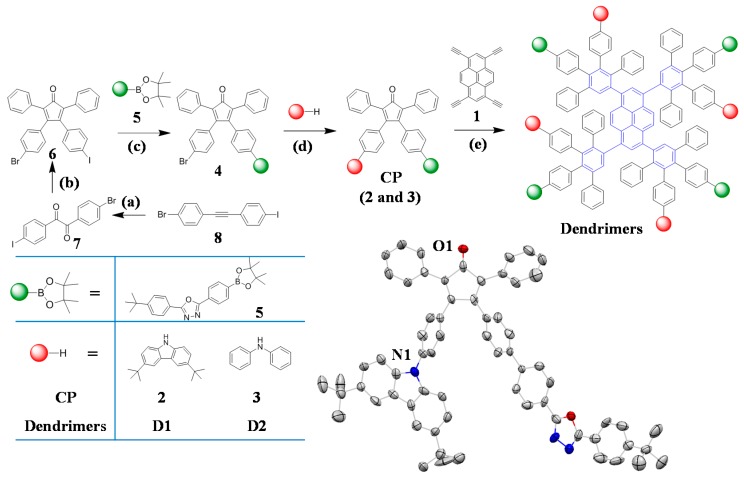
Synthetic routes of donor-acceptor dendrimers and crystal structure of **CAB**- and **PBD**-functionalized CP **2** (bottom right). *Reagents and Conditions*: (a) I_2_, DMSO, 155 °C, 24 h, 89%; (b) 1,3-diphenylacetone, KOH, EtOH, 80 °C, 1 h, 76%; (c) Pd(PPh_3_)_4_, K_2_CO_3_, tetrabutylammonium bromide, toluene, H_2_O, 48 h, 80 °C, 100%; (d) Pd_2_(dba)_3_, t-BuONa, (t-Bu)_3_P, toluene, 24 h, 110 °C, 78% for **2**, 75 °C, 39% for **3**; (e) *O*-xylene, 130 °C, 24 h, 50% for **D1**, 45% for **D2**.

The synthesis of donor-acceptor dendrimers needs a bromo- and iodo-functionalized tetraphenylcyclopentadienone (CP) **6** (Scheme 1). Compound **6** was prepared in two steps from 1-(4-bromophenyl)-2-(4-iodophenyl)ethyne (**8**) which in turn was made in high yield (67%) by a one-pot double elimination process [42,43]. Compound **8** was oxidized by I_2_ and DMSO to give 1-(4-iodophenyl)-2-(4-bromophenyl) diketone (**7**) in high yield (89%). CP **6** was then obtained (in 76%) from **7** and 1,3-diphenylacetone through a Knoevenagel condensation. The donor- acceptor CPs **2** and **3** were prepared in two steps. First, a Suzuki coupling between the bromo- and iodo-functionalized CP **6** and **PBD**-boronic ester **5** at 80 °C for 48 h furnished a bromo- and **PBD**-functionalized CP (**4**) quantitatively. The high selectivity of the Suzuki coupling was a key for the next step, in which Buchwald-Hartwig reactions between CP **4** and 3,6-di(*tert*-butyl)-9*H*-carbazole or diphenylamine were performed to form CP **2** and **3** in moderate to high yields (39% and 78%, respectively). Finally, the dendrimers were prepared in moderate yields by a Diels-Alder reaction between 1,3,6,8-tetraethynylpyrene (**1**) and the CPs. Consequently, with the bromo- and iodo-functionalized CP **6** available, it is feasible to produce other functional materials including small molecules, dendrimers and polymers for versatile applications and fundamental research, such as dendrimers with hydrophilic and hydrophobic segments in the periphery as drug carriers into cells due to their amphiphilic solubility [44].

The crystal structure of the **CAB**- and **PBD**-functionalized CP **2** (Scheme 1) showed that the torsion angles between the central pentagon and phenyl groups at the 3,4-positions were 53° and 48° respectively due to the steric hindrance (see Figure S8). The corresponding angles for the **PBD** segment in this CP were 30°, 11° and 14° respectively, which were a little higher than the reported bare **PBD** single crystal results, namely 26°, 8° and 4° [45]. This suggests that the conjugation of the **PBD** moiety in the CP is somewhat reduced as compared with **PBD**.

Both dendrimers are readily soluble (≥8 g/L) in organic solvents, e.g., DCM, THF and toluene. They were purified by silica gel flash column and GPC chromatography methods and their characterization was accomplished by a combination of ^1^H-NMR and ^13^C-NMR spectroscopy, MALDI-TOF mass and high-resolution mass spectrometry (HRMS), UV-vis absorption and (time-resolved) photoluminescence (PL) spectroscopy, transient absorption spectroscopy and cyclic voltammetry. ^1^H-NMR spectroscopy could distinguish some special protons of the dendrimers (see Figures S1–S3). For example, in **D1**, the protons H_a_ and H_b_ which were closest to 1,3,4-oxidazole groups located at the lowest fields due to the strong electron-withdrawing effect of the 1,3,4-oxidazole moiety. Proton signals from the pyrene (H_d_ and H_e_) and 4- and 5-positons (H_c_) on the carbazole at low field overlapped with the H_b_ proton (8.09–8.05 ppm). MALDI-TOF mass spectra only showed the molecular ion peaks of the respective dendrimers (Figure S4) and the HRMS spectra of **D1** exhibited clear isotope patterns of the molecular ion, consistent with the calculated result. Consequently, the structures of the desired dendrimers were firmly proven.

### 2.2. Photophysical Properties

**Figure 2:**
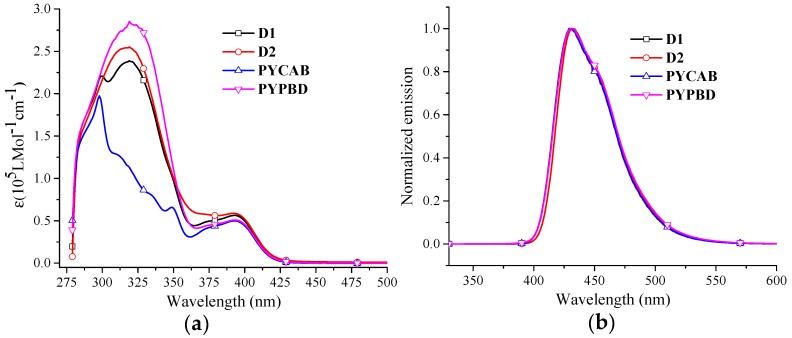
UV-vis absorption (**a**) and PL spectra of dendrimers (**b**), excited at 299 nm for **PYCAB**, 319 nm for the others (10^−5^ M, toluene).

The optical absorption spectra of these dendrimers generally features two major bands (Figure 2). One with characteristic shape and significant intensity (ε: 230,000–250,000 L·mol^−1^·cm^−1^) is attributed to the energy transitions of PCs and polyphenylenes [37,38,39]. For example, in **D1**, the band with a maximum at 299 nm is characteristic of the **CAB** absorption [37], also consistent with the strongest absorption peak of **PYCAB** (a pyrene-cored polyphenylene dendrimer with **CAB** in the periphery, see Supplementary Materials) [24]. The band with a maximum at 319 nm can be assigned to the absorption of **PBD** [38], similar to that of **PYPBD** (a pyrene-cored polyphenylene dendrimer with **PBD**s in the periphery, see Scheme S1). The other band (λ_max_: 392 nm, ε: ~50,000 L·mol^−1^·cm^−1^) is much sharper and originates from π-π* transitions of the pyrene core [24,40,41].

The photoluminescence spectra (PL) of these dendrimers showed that the emission of the donor-acceptor dendrimers, **PYCAB** and **PYPBD** were all identical (λ_max_: ~431 nm and a weak shoulder: ~455 nm) both for excitation of the surface and the core groups (Figure 2), revealing that the emission of the donor-acceptor dendrimers can be attributed to an efficient surface-to-core energy transfer [24,40,41]. In thin films, both donor-acceptor dendrimers and **PYPBD** give moderate bathochromic shifts (10–20 nm) (Table 1), assigned to the intermolecular interactions among the cores or between the cores and PCs due to inadequate protection of the cores by the polyphenylene dendrons for the first-generation dendrimers [24,40].

The photoluminescence quantum yields (PLQYs) are generally rather high, regardless if the excitation is done via the core (0.8~0.9) or the surface moieties (0.6~0.8) (Table 1). The optical energy gaps of all dendrimers are in the range of 2.95 eV (within measurement errors) (Table 1).

**Figure 3:**
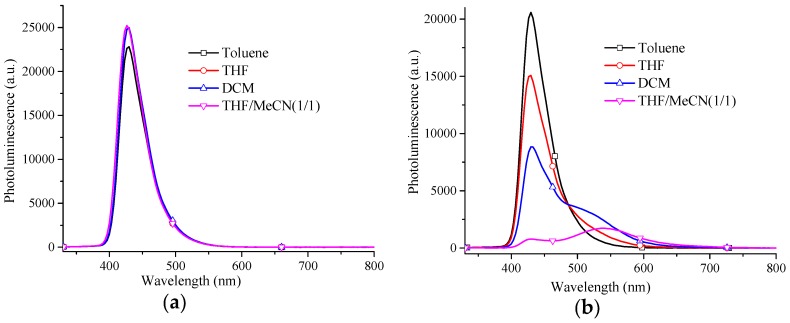
PL spectra of **D1** (**a**) and **D2** (**b**) in different solvents (excited at 319 nm, 3.5 × 10^−6^ M (**a**); 4.0 × 10^−6^ M (**b**)).

Since donor-acceptor characteristics (ICT) are expected for **D1** and **D2** in polar solvents [28,46], absorption and emission spectra of these materials were measured in different solvents, i.e., toluene, THF, CH_2_Cl_2_, THF/acetonitrile (1/1) and acetonitrile (poor solubility). There were no noticeable changes in most solvents (see Figure S10), except for small hypsochromic shifts in THF/acetonitrile solution in case of both dendrimers. This suggests that the ground states of both dendrimers are more polar than their excited states [36]. The PL results for **D1** failed to reveal bathochromic shifts of the emission or notable fluorescence quenching in solvents of different polarity, also independent of the excitation at the surface or the core (Figure 3 and Figure S11). There is thus, no ICT. The emission of **D2**, on the other hand, apparently displayed a significant bathochromic shift and fluorescent quenching with increasing solvent polarity (Figure 3 and Figure S11). Interestingly, both emission from the core and the ICT state coexisted, and their relative intensities were tunable depending on the polarity of the environment. Thus, in THF/acetonitrile, one fluorescence peak at 431 nm and the other one at 538 nm contributed to a yellow-white emission.

**Figure 4:**
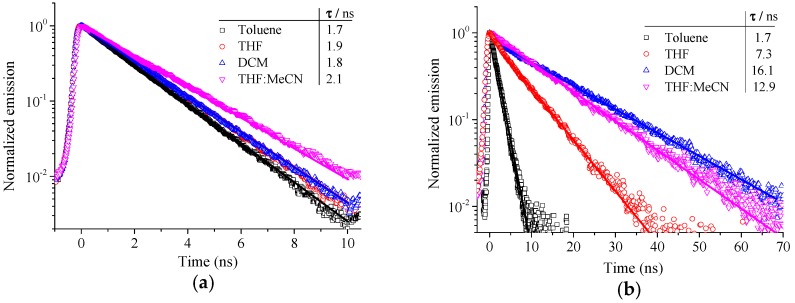
PL decay (open symbols) of **D1** (**a**) and **D2** (**b**) tracked from 425–445 nm and 490–510 nm, respectively. Solid lines represent fits with a monoexponential decay function.

To further investigate the findings of the steady-state spectroscopy, time-resolved photoluminescence (TRPL) and transient absorption (TA) spectroscopy experiments were conducted. Again, the emission of **D1** did not change with solvent polarity (see Figure S13) and the decay dynamics remained rather unaffected by the solvent as shown in Figure 4a with inverse decay rates of ~2 ns. On the contrary, as depicted in Figure 4b, the effect of the solvent on the decay dynamics of **D2** was very pronounced. A monoexponential fit to the data revealed that the lifetime increased from 1.7 ns in toluene to 12.9 ns and 16.1 ns in THF:MeCN and CH_2_Cl_2_, respectively. The spectral shifts found in TRPL experiments were consistent with the steady-state PL experiments (see Figure S14). The emission of **D2** in toluene was similar to that of **D1**, which did not shift and remained constant with the emission decay. A slight redshift was observed for THF. **D2** in DCM showed an intermediate shift of the emission. The maximum was still at around 435 nm, however, a shoulder developed at 510 nm. **D2** in THF:MeCN exhibited the most pronounced bathochromic shift with respect to toluene which was in line with the steady-state fluorescence measurements. Here, the maximum was at 510 nm (ICT emission) and the signal at 435 nm (core emission) was present only after excitation. The core emission decayed very quickly and consequently the shoulder disappeared at longer delay times. The bathochromic shift in combination with the prolonged lifetime suggested the formation of an ICT state in **D2** in solvents with higher polarities. In order to better track the early decay behavior, **D2** in THF:MeCN was additionally investigated on the picosecond timescale (see Figure S15). A pure core emission (λ_max_: ~425 nm) was detected at the beginning, but a broad band centered around 510 nm (ICT emission) developed quickly. For example, at 60 ps, both core and ICT emission were detected and at 420 ps, the ICT emission became more pronounced while the core emission was less noticeable. In addition, a monoexponential fit to the decay of the singlet emission resulted in an inverse decay rate of 15.5 ps which was the time for the S_1_→ICT conversion. These findings clearly manifest the coexistence of core and ICT emission and their transformations. They also indicate that the dual emission for **D2** in polar solvents is due to the transformation of localized excited state (S1) into the ICT state which is much stabilized in highly polar environments (see Scheme S2). Similar S_1_→ICT conversions were also discovered in some small molecules [47,48]. Whether the ICT state is due to a more twisted or more planarized molecular structure than in the ground state [34,49], is not investigated here. One might anticipate, however, that the **TPA** moieties of **D2** may undergo slight rotations with respect to the core in the ICT state due to the strong steric hindrance within the molecule (see Figure S18).

**Figure 5:**
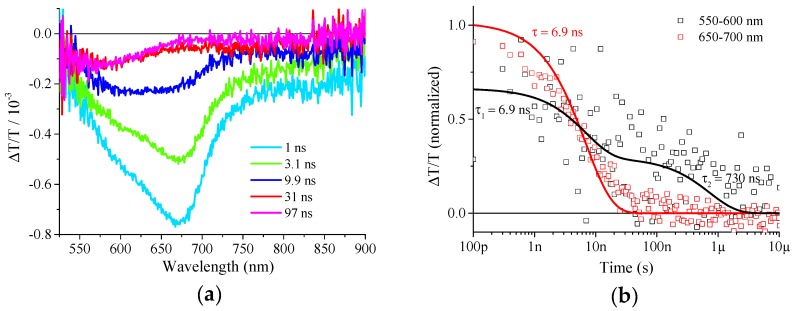
Transient spectra at different delay times after excitation with 355 nm (**a**) and decay dynamics tracked from 550–600 nm and 650–700 nm (**b**).

To clarify whether the observed redshift and prolonged lifetime in emission spectroscopy are related to ICT formation, transient absorption (TA) spectroscopy was performed on **D2** dissolved in THF:MeCN (Figure 5). Directly after excitation the TA spectrum was dominated by a peak at 670 nm which decayed with an inverse decay rate of 6.9 ns. This was shorter compared to the inverse decay rate observed in the TRPL experiment where an inverse decay rate of ~13 ns was observed. However, a laser with a pulse duration of ~700 ps was used for TA experiments compared to ~100 fs in TRPL which could contribute to slightly varying lifetimes. Amthor et al. demonstrated that the radical cation absorption of a similar triarylamine was found at 14990 cm^−1^ and 17360 cm^−1^ which corresponded to 576 nm and 667 nm, respectively [50]. This is in very good agreement with the signal observed in TA experiments and therefore we can clearly assign the species witnessed in **D2** in THF:MeCN to the formation of an ICT.

On a timescale of tens of nanoseconds, the TA spectrum shifts to a feature peaking at 580 nm which further decays with an inverse decay rate of 730 ns. According to Amthor et al. the two transitions can be assigned to the transition D_0_(A)→D_1_ (A) and D_0_(A)→D_2_ (A) for the low and high energy transitions, respectively [50]. However, the reason for the change in the transition probability of the radical cation to the first and second excited state with time remains unclear. Alternatively, the ICT might recombine to form a triplet if electrons in charge-transfer states lose their spin correlation. This effect has been observed for CT states in organic photovoltaics for blends of electron donor and acceptor [51,52]. Such a conclusion would be in line with the rather long decay time extending to 1 µs. To summarize the results of time-resolved spectroscopy, the prolonged lifetime in TRPL and the photoinduced absorption observed in TA experiments clearly prove the formation of an ICT state.

**Figure 6:**
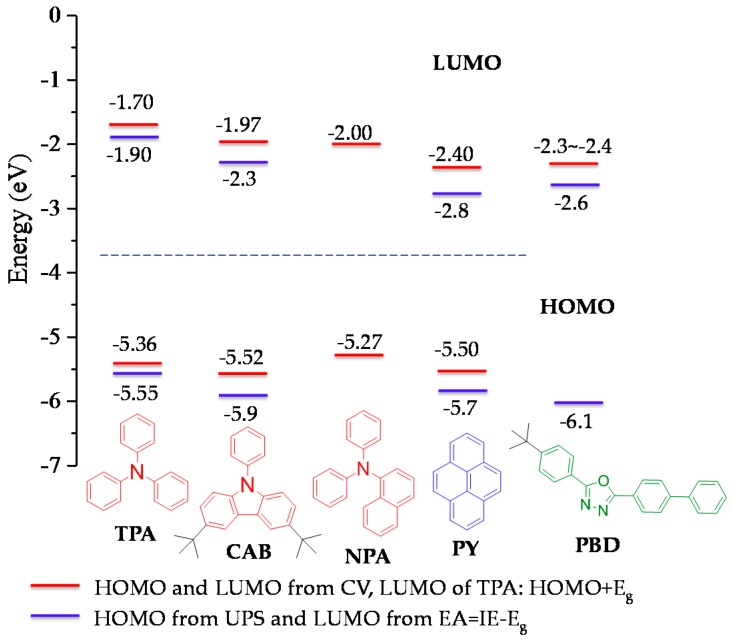
HOMO and LUMO levels of PCs and pyrene estimated from CV of dendrimers and literature (red) [24,59], from reported UPS measurements [23,37,60,61].

The reason for the lack of ICT in **D1** and noticeable ICT in **D2** in polar environment is related to the electron donating ability of **CAB/TPA** to the core. The PL spectra of triphenylamine- functionalized, pyrene-cored polyphenylene dendrimer (**PYTPA**) were measured in solvents of different polarities (see Figure S16). The experiment disclosed that **PYTPA** underwent notable ICT. It became more pronounced in highly polar solvents and similar to **D2**, **PYTPA** displayed notable dual emission in THF:CH_3_CN with the band shapes and positions nearly identical to those observed for **D2**. Accordingly, the ICT in **D2** is mainly due to the electron-donation of **TPA** to the core. This is consistent with the higher HOMO level of **TPA** (~−5.34 eV) than that of the core (~−5.50 eV) together with the lower LUMO of the core (~−2.40 eV) than that of **TPA** (~−1.70 eV), which facilitates **TPA**-to-core electron transfer (Figure 6, Table 1). ICT phenomena induced by amine-to-pyrene electron transfer were reported before [53,54,55,56]. Dual emission was also detected for 1-(*p*-amino-phenyl)pyrene under acidic conditions [57]. For comparison, the polarity-dependent PL properties of **PYCAB** were also studied (see Figure S17). The results suggested that no ICT occurred in **PYCAB** similar as **D1**. Therefore, it is postulated that the absence of ICT in **D1** is due to the lack of electron donation from **CAB** to the core, consistent with the similar HOMO levels between **CAB** (−5.52 eV) and the core (−5.50) (Figure 6, Table 1). The electron accepting effect of **PBD** in both donor-acceptor dendrimers was probably minor because the LUMO of **PBD** (−2.30~−2.40 eV) in both dendrimers was slightly higher than that of the core (~−2.40 eV) (Figure 6).

To the best of our knowledge, **D1** represents the first known donor-acceptor dendrimer which does not show ICT. It also gives an insight to design new donor-acceptor or donor-acceptor-acceptor systems with minimized ICT by tuning the HOMO/LUMO of the components. As far as we know, **D2** and **PYTPA** are the first reported dendrimers with dual emission. **D1** as a donor-acceptor material without ICT may promise a good blue emitter in OLEDs. The observed dual emission in **D2** and **PYTPA** under polar environments may give an insight into designing new emitters [58].

### 2.3. Electrochemistry

**Figure 7:**
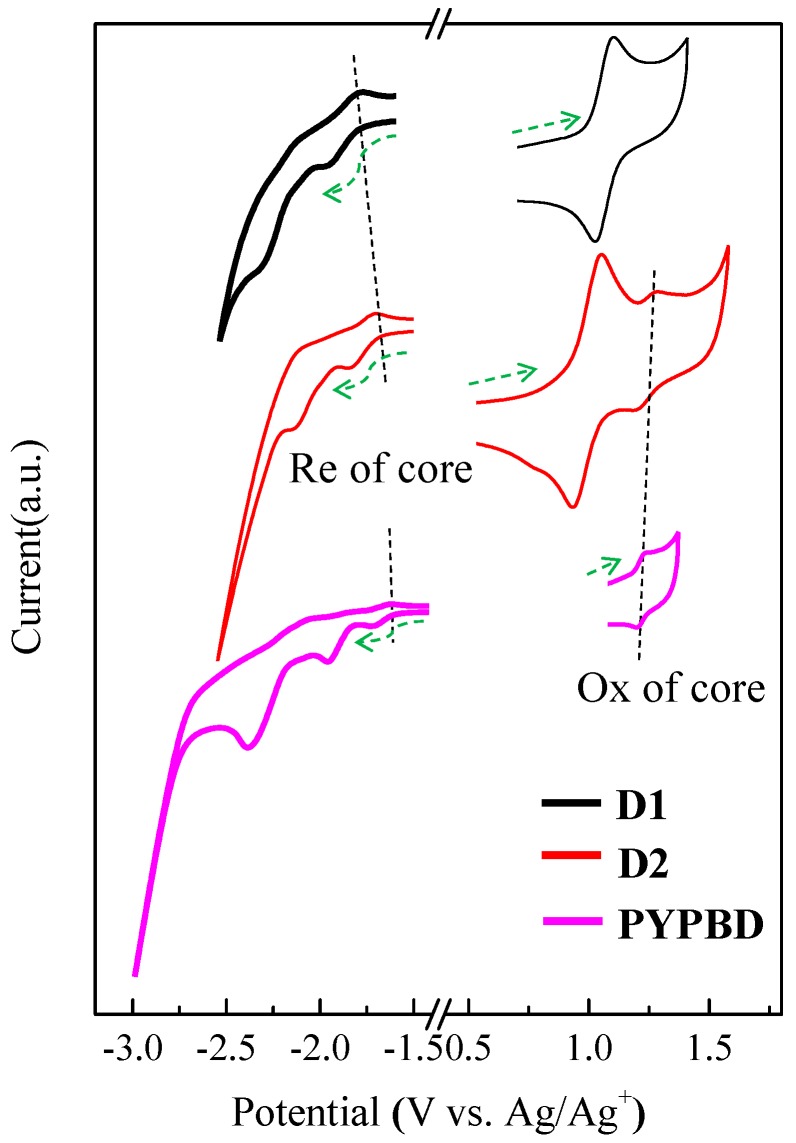
CV curves of the redox of **D1**, **D2** and **PYPBD** (arrow: scan direction).

As expected, CV curves of these dendrimers revealed the redox waves of the core and reduction/oxidation of the PCs (Figure 7). For example, the oxidation peak of the core located at around 1.2 V (vs. Ag/Ag^+^) as demonstrated for **D2** and **PYPBD**. For **D1**, the oxidation of the core and **CAB**s merged as one due to their very close oxidation potentials [24]. The reduction of these dendrimers (~−1.7 V vs. Ag/Ag^+^) started from the core, similar to previously reported pyrene-cored polyphenylene dendrimers [24]. The LUMOs of the donor-acceptor dendrimers were both around −2.4 eV (considering the measurement errors) by comparing with ferrocene (Table 1), similar as other pyrene-cored polyphenylene dendrimers [24].

The LUMO of **PYPBD** (~−2.5 eV) was slightly lower, probably due to electron-withdrawing effects of **PBD**s. As to the redox of PCs, the reduction of **PBD** appeared following the core. For example, there existed an enhancement in the core reduction for **D2** and **PYPBD**, due to an overlap between the reduction wave of the core and **PBD**. The LUMO of **PBD** was estimated to be between −2.30 and −2.40 eV, slightly higher than bare **PBD** (−2.40 eV) [62], considering the results of first and second reduction onset of **D2**.

Moreover, the oxidations of the **TPA**s and **CAB**s contributed to the major oxidations of the donor-acceptor dendrimers. The HOMOs of **TPA** and **CAB** were calculated to be −5.34 and −5.52 eV, respectively, corresponding to the HOMOs of the dendrimers. These values are very close to the HOMO of bare **CAB** (−5.61 eV) [37] and **TPA** (−5.35 eV) [59] (considering the measurement errors), suggesting little conjugation interactions between the PCs and the core in these dendrimers due to the high torsion angles between the PCs and the four phenyl groups bonding to pyrene, consistent with the crystal structure of compound **2** (Figure S8). The HOMO of the core was deduced to be around −5.50 eV from the oxidation wave of **PYPBD**. The oxidation of **PBD** was beyond the detection limit. This is consistent with the HOMO level of **PBD** obtained from ultraviolet photoemission spectroscopy (UPS), which is much lower than for other PCs (Figure 6).

Accordingly, **D1** with a donor-acceptor surface (HOMO: −5.52 eV, LUMO: −2.30~−2.40 eV) and **D2** with a donor-acceptor surface (HOMO: −5.34 eV, LUMO: −2.30~−2.40 eV) should reduce both hole and electron injection barriers from the electrodes and facilitate both hole and electron transport in devices (Figure 6). Based on their photophysical properties, it indicates that the donor-acceptor dendrimers exhibit efficient surface-to-core energy transfer and pure blue emission with high PLQYs and **D1** shows no ICT. In addition, electrochemistry results demonstrate that the donor-acceptor dendrimers could facilitate both hole and electron injection. As a result, the donor-acceptor dendrimers and **PYPBD** (for comparison) were tested in OLEDs.

### 2.4. OLED Device Performances

Due to the nonexistence of ICT in **D1**, it was employed in single-, two- and three-layer devices. The single-layer devices had an ITO/PEDOT:PSS/dendrimer/Ca/Al configuration, where the dendrimer was spin-coated on the PEDOT:PSS surface in THF, toluene or chlorobenzene solution (Supplementary Materials). As depicted in Table 2, the single-layer device has a pure blue emission and low current efficiency and luminance and cannot render a high-performance single-layer device. However, this device in comparison with the single-layer device of **PYPBD** has a remarkably reduced onset voltage due to the donor-acceptor surface of **D1**.

This dendrimer was optimized in a two-layer device of ITO/PEDOT:PSS/TFB/dendrimer/Ca/Al, where poly(9,9-dioctylfluorene-co-*N*-(4-butylphenyl)-diphenylamine) (TFB) worked as hole-transporting material (HOMO: −5.30 eV, LUMO: −2.10 eV) [63]. The dendrimer layer was spin-coated on a TFB surface (Supplementary Materials). A better performance was achieved with the highest current efficiency of 0.13 cd/A and luminance of 1866 cd/m^2^, probably due to better charge confinement in the emitting layer with the help of TFB. It was further optimized in a three-layer device with the structure of ITO/PEDOT:PSS/TFB/dendrimer/PEGPF/Ca/Al, where 9,9-bis(3-(5′,6′- bis(4-(polyethyleneglycol)phenyl)-[1,1′:4′,1″-terphenyl]-2′-yl)propyl)-9′,9′-dioctyl-2,7-polyfluorene (**PEGPF**) worked as electron transporting layer [64]. The device performance was enhanced. The current efficiency could reach 0.21 cd/A and the highest luminance was 2700 cd/m^2^. This was probably due to the enhanced electron injection compared with a two-layer device and a better charge confinement within the emitting layer. In general, the emission in all three devices came solely from the core of the dendrimer based on their very similar CIE coordinates and their peak emission all at 444 nm (see Figure S20), nearly the same as the thin film emission of **D1** (λ_max_ = 446 nm).

**D2** was tested in a two-layer device. However, the device performance in current efficiency, luminance and CIE coordinate was not as good as that of **D1** (Table 2) and another small peak appeared at around 620 nm in the electroluminescence spectra (see Figure S20), possibly due to the ICT effect in **D2** induced by the electrical field in the device. As a result, no further device investigation was conducted. **PYPBD** was tested in two- and three-layer devices as well and in general, it could reach a comparable current efficiency as **D1** but the onset voltage, luminance and CIE coordinate were not as good as **D1**. Consequently, **D1** represents the best among the three materials in blue OLEDs, probably due to a possibly improved bipolar charge transport and absence of ICT.

**Figure 8:**
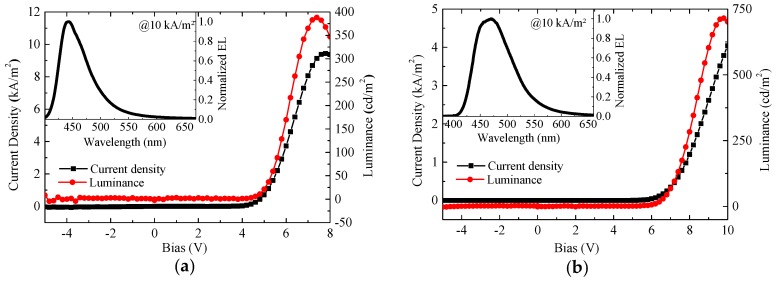
Voltage-current-luminance characteristics of **D1** (**a**) and **PYNPA** (**b**) in single-layer devices (inset: the electroluminescence spectra).

However, even though **D1** contains both donor and acceptor components, the overall device performances are not notably improved compared with the previously reported dendrimers, e.g., **PYNPA** (single layer performance: 0.24 cd/A and 715 cd/m^2^, three-layer performance: 0.45 cd/A and 3726 cd/m^2^) (see Table S2) [24]. This can be tentatively explained by the less-effective hole and electron recombinations in **D1**. As depicted in Figure 8, the single layer device of **D1** displays much higher current density than **PYNPA** (e.g., 9 kA/m^2^ vs. 1 kA/m^2^ at 8 V). This reflects that there are many more charges in the device of **D1** than that of **PYNPA** due to the reduced charge injection barrier or increased charge carrier mobilities or both in the donor-acceptor dendrimer. However, these charges do not recombine effectively in **D1**. In the **D1**-based device, electrons were probably transported from the LUMOs of **PBD** moieties into the LUMOs of **CAB** cations (holes) to form excitons through intermolecular hopping between nearby **PBD** and **CAB** (the energy barrier between LUMO of **PBD** and LUMO of **CAB**: ~0.3 eV, see Figure 6). For **PYNPA**, on the other hand, electrons hopping along the HOMOs and LUMOs of **NPA**s had energy barriers of zero theoretically. As a result, **D1** is less effective to generate excitons than **PYNPA**, partly due to the energy barrier between **PBD** and **CAB** for electron transport.

## 3. Materials and Methods

All chemicals and solvents were purchased from commercial sources and used as received except where noted. Reactions were all conducted under argon atmosphere. ^1^H-NMR and ^13^C-NMR spectra were recorded on AMX 250, AC 300, AMX 500, and AMX 700 NMR spectrometers (Bruker Bio-Spin GmbH, Silberstreifen, Rheinstetten, Germany). The solvents for NMRs were CD_2_Cl_2_ with the reference peak at 5.32 ppm (^1^H) and 53.84 ppm (^13^C) and THF-d_8_ with the reference peak at 1.72 ppm (^1^H) and 25.31 ppm (^13^C). Field desorption (FD) mass spectra were recorded on a ZAB 2-SE-FDP using 8 kV accelerating voltage (*VG* Masslab, Crewe Road, M23 9BE, *Manchester*, UK). MALDI-TOF mass spectra were recorded by a Bruker Reflex II spectrometer (BRUKER DALTONIK GmbH, Life Sciences & Chemical Analysis, Fahrenheitstr. 4, D-28359 Bremen, Germany) with fullerene as the reference and dithranol as the matrix. UV-Vis absorption spectra were measured by a Varian Cary 4000 UV/Vis spectrometer (Varian Inc. Palo Alto, CA, USA) using quartz cells with path length of 1 cm. Fluorescence spectra were recorded with a Fluorolog 2 spectrometer (Horiba, Kyoto, Japan). Cyclic Voltammetry (CV) measurements were conducted on a computer-controlled Biopontiostat CH 1760 (CH Instruments, Inc. Glasgow, Scotland) using a three electrodes system in which a Pt wire, a silver wire, and a glassy carbon electrode were used as counter, reference and working electrode respectively. The measurements were conducted in 0.1 M tetrabutylammonium hexafluorophosphate (TBAPF) solution under an argon environment with a scan rate of 100 mV/s at room temperature. The solvents were DCM for the oxidation part and THF for the reduction part. Ferrocene (Fc) worked as the internal reference throughout the measurements. The calculations of HOMO and LUMO energies using CV were based on the following equations, HOMO (eV) = −E_ox_^onset^ + E_Fc_^onset^ – 4.80 and LUMO (eV) = −E_red_^onset^ + E_Fc_^onset^ – 4.80 where E_ox_^onset^, E_Fc_^onset^ and E_red_^onset^ mean the onset oxidation potential of dendrimers, the onset oxidation potential of ferrocene and the onset reduction potential of dendrimers compared to a silver reference electrode. The calculated isotope distributions of the molecular mass of dendrimers were conducted with mMass software.

Time-resolved photoluminescence (TR-PL) spectra were taken with a C4742 streak camera system (Hamamatsu Photonics, Hamamatsu, Japan) in fast/slow sweep mode. Excitation pulses at 400 nm were provided by frequency doubling the output of a commercial femtosecond oscillator (Coherent Vitesse, Santa Clara, CA, USA) or amplifier laser system (Coherent LIBRA-HE, Santa Clara, CA, USA), for fast/slow sweep, respectively.

Transient absorption (TA) measurements were performed with a home-built pump-probe setup. To generate white light in the visible the output of a commercial titanium:sapphire amplifier (Coherent LIBRA-HE, 3.5 mJ, 1 kHz, 100 fs) was used to pump a commercial optical parametric amplifier (Coherent OPerA Solo). The parametric amplifier (OPA) was used to generate the seed beam of 1300 nm for white-light generation. The 1300 nm seed of a few µJ was focused into a c-cut 3 mm thick sapphire window for white-light generation. Mostly reflective elements were used to guide the probe beam to the sample to minimize chirp. The excitation pulse was provided by an actively Q-switched Nd:YVO4 laser (MOPA, AOT Ltd., Essex, UK) at 355 nm with a resolution of 600 ps. The delay between pump and probe was controlled by an electronic delay generator (DG535, Stanford Research Systems, Sunnyvale, CA, USA). The white-light pulses were dispersed onto a linear silicon photodiode array, which was read out at 1 kHz by home-built electronics. Adjacent diode readings corresponding to the transmission of the sample after an excitation pulse and without an excitation pulse were used to calculate ΔT/T. TA measurements were performed at room temperature. The methods for single crystal structure extraction, DFT calculations, fabrication and characterization of OLEDs and synthesis are described in detail in the Supplementary Materials.

## 4. Conclusions

Two donor-acceptor and blue-light-emitting dendrimers with efficient surface-to-core energy transfers were synthesized based on a new bromo- and iodo-functionalized CP **6**. This method paves the way for the development of diverse multifunctional materials. **D1** showed a pure core emission without ICT under polar conditions due to the poor electron donation from **CAB** to the core. To the best of our knowledge, **D1** is the first reported donor-acceptor and non-ICT dendrimer, which is also an important example for designing new donor-acceptor or donor-acceptor-acceptor systems with minimized ICT in the future. **D2** and **PYTPA** exhibited dual emission from both the core and ICT state in polar solvents due to **TPA**-to-core electron transfer, which are the first reported dendrimers showing dual emission. The OLED performances of the donor-acceptor dendrimers and **PYPBD** suggest that **D1** is the best among the three, with a pure blue emission, low onset voltage and high luminance due to its assumed to be improved bipolar charge transport and non-ICT properties. As far as we know, **D1** also represents the first pure blue dendrimer emitter with bipolar charge transport capabilities and surface-to-core energy transfer in OLEDs. However, when comparing the performance of the donor-acceptor dendrimers with light emitting dendrimers bearing only a single type of transport moiety on the surface, it is obvious that we could not significantly improve the performance in a simple device geometry as presented here. This is attributed to the fact that exciton formation from the geometrically rather distant transport groups on one chromophore is not favored in the structures we have presented here. To overcome this problem, the size/generation of the dendrimers must be extended and a functional moiety which allows for balanced charge transport and efficient exciton formation needs to be introduced on the outer shell. Putting the results of this work in context with the currently occurring rapid developments in the field of TADF-emitters and -hosts, it is easy to envisage a dendrimer design where a suitable bipolar moiety functions as a host matrix in the outer shell and a respectively tuned TADF core is placed in the center. However, as demonstrated in this work, certain care has to be taken in the design of the host-moiety as ICT would be detrimental to device performance.

## Figures and Tables

**Table 1:** Photophysical and electrochemical properties of dendrimers.

Dendrimer	λ_ab_ (nm)		λ_em_ (nm)		PLQY ^a^		HOMO (eV) ^b^	LUMO (eV) ^b^	Eg (eV) ^c^
	Sol	Film	Sol	Film	Excitation				
					Surface	Core			
**D1**	299, 319, 392	300, 316, 391	431	446	0.80	0.93	−5.52	−2.45	2.95
**D2**	319, 392	313, 393	432	440	0.59	0.85	−5.34	−2.44	2.95
**PYPBD**	320, 392	320, 393	431	453	0.66	0.78	−5.50	−2.52	2.97

^a^ Measured in THF solution with quinine sulfate in 0.5 M H_2_SO_4_ solution as the standard, error margins: ±1.5%; ^b^ calculated from CV by comparing the first redox onset of dendrimers and the oxidation onset of ferrocene; ^c^ calculated from the absorption edge of the longest wavelength band.

**Table 2:** Device performances of dendrimers.

Dendrimer	V_on_ (V)	Η (cd/A)	L_max_ (cd/m^2^)	CIE (*x*, *y*)
**D1**	4	0.048	388	0.16, 0.10 ^a^
	5.5	0.13	1866	0.16, 0.12 ^b^
	4.4	0.21	2701	0.16, 0.12 ^c^
**D2**	4.8	0.02	203	0.20, 0.18 ^b^
**PYPBD**	9.7	0.04	277	0.18, 0.18 ^a^
	7	0.17	854	0.18, 0.18 ^b^
	7	0.24	1288	0.18, 0.18 ^c^
**PYNPA**	5.2	0.24	715	0.16, 0.20 ^a^

Device structure: ^a^ ITO/PEDOT:PSS/dendrimer/Ca/Al; ^b^ ITO/PEDOT:PSS/TFB/dendrimer/Ca/Al; ^c^ ITO/PEDOT:PSS/TFB/dendrimer/PEGPF/Ca/Al.

## Supplementary Materials

The following are available online at: http://www.mdpi.com/1420-3049/21/10/1400/s1: experimental details, NMR and MALDI-TOF mass spectra, crystal structure, absorption and photoluminescence spectra (steady state and time resolved) in different solvents, CV curves and electroluminescence spectra.
