# Supplementary material for: Blue Light Emitting Polyphenylene Dendrimers with Bipolar Charge Transport Moieties
Source: Molecules. 2016 Oct 20;21(10):1400. doi: 10.3390/molecules21101400 (PMC6273451; doi:10.3390/molecules21101400)
Supplement: Supplementary file 1 [file molecules-21-01400-s001.pdf]

Supplementary Materials: Blue Light Emitting Polyphenylene Dendrimers with Bipolar Charge Transport Moieties

Guang Zhang, Manuel Auer-Berger, Dominik W. Gehrig, Paul W. M. Blom, Martin Baumgarten, Dieter Schollmeyer, E J.W. List-Kratochvil and Klaus Müllen

1. Description of X-ray analysis

The X-ray intensity data were measured on a Bruker SMART APEX2 CCD-based X-ray diffractometer system (Bruker AXS GmbH, Östliche Rheinbrückenstr. 49, 76187 Karlsruhe, Germany) equipped with a Mo-target X-ray tube. The data frames were collected using the program APEX2 and processed using the program SAINT routine within APEX2. The data were corrected for absorption based on the multi-scan technique as implemented in SADABS. Structure solution and refinement were performed using SHELT-2014. Crystal of compound **2** contains one molecule disordered molecule $CHCl_3$.

2. DFT calculations

The DFT calculations for ground state geometry optimizations were performed using PC controlled Gaussian software (Gaussian 09, Gaussian Inc., Wallingford, CT, USA) with B3LYP hybrid and 6-31g(d) basis set.

3. Fabrication and Characterization of OLEDs

For the fabrication of single- and multilayer-devices all indium tin oxide covered glass substrates were cleaned mechanically by the use of acetone and isopropanol. The substrates were subjected to subsequent supersonic cleaning steps in deionized water, toluene and isopropanol. Prior to the depostition of poly(3,4-ethylenedioxythiophene)-polystyrenesulfonic acid (Baytron P VPAI 4083) (PEDOT:PSS) from H. C. Starck as a first polymeric layer, to enhance the wettability of PEDOT:PSS on the substrate, the ITO substrates were subdued to a dry cleaning procedure in oxygen plasma. PEDOT:PSS was spin-cast onto the as prepared substrates under ambient conditions and dried according to the specifications under dynamic vacuum with a pressure less than 1×10^{-5} mbar. For single layer DLEDs the emissive layer was spin cast from 8 $mg \cdot mL^{-1}$ toluene solutions. After spin coating the samples were annealed for 1 h under high vacuum ($p \leq 1 \times 10^{-5}$ mbar) at 70 °C. The thicknesses of the emissive layers were determined to be 50 nm with a mean error of less than 10%. To fabricate triple-layer-OLEDs, TFB layers were deposited by spin-coating from a 15 $mg \cdot mL^{-1}$ solution under inert conditions and dried in argon at 200 °C for 2 h. Afterwards, the substrates were cooled to room temperature on a cold surface. To remove the soluble parts of the annealed polymer the TFB layer was spin-rinsed by pure toluene resulting in a final layer thickness of 5 nm with good reproducibility as long as annealing temperature and time were kept constant. Emissive layers were spin-cast onto prepared TFB films from an 8 $mg \cdot mL^{-1}$ toluene solution and dried at 80 °C for 2 h in vacuum ($p \leq 1 \times 10^{-5}$ mbar) resulting in layer thicknesses of 75 nm. Prior to depositing PEGPF in the case of triple-layer devices, the TFB/dendrimer-substrates were spin-rinsed by pure methanol. Afterwards PEGPF dissolved in methanol was applied by spin-coating at a polymer content of 1 $mg \cdot mL^{-1}$. The layer was annealed at 70 °C for 1h under high vacuum ($p \leq 1 \times 10^{-5}$ mbar) and exhibited a thickness of 10 nm, which was well controllable for spinning speeds above 1500 rpm. After polymer deposition 1 nm of Cs_2CO_3 and 100 nm Al as cathode materials were deposited thermally through a shadow mask forming a device area of 9 mm^2 in a custom made vapor deposition unit from tungsten boats at an initial base pressure less than 1×10^{-6} mbar. Current/luminance/voltage (I-L-V) characteristics were recorded in a customized setup. To determine the I-V characteristics a Keithley 2612A source measure unit was used and a Keithley 6485 Picoammeter (Keithley Instruments, Inc.

Corporate Headquarters 28775 Aurora Road, Cleveland, OH, USA) using a calibrated photodiode was employed for recording the luminance.

4. Synthetic Procedures

1,3,6,8-Tetraethynylpyrene(**1**), [1] 2-(4-tert-butylphenyl)-5-(4-(4,4,5,5-tetramethyl-1,3,2-dioxaborolan-2-yl)phenyl)-1,3,4-oxadiazole(**5**) [2], 1-(4-bromophenyl)-2-(4-iodophenyl)ethyn(**8**) [3,4], 3,4-bis[4-(4,4,5,5-tetramethyl-1,3,2-dioxaborolan-2-yl)phenyl]-2,5-diphenylcylcopentadienone (**10**) [5] and 2-bromophenyl-5-(*p*-tertbutyl-phenyl)-1,3,4-oxadiazole (**11**) [6] were synthesized according to the published procedures.

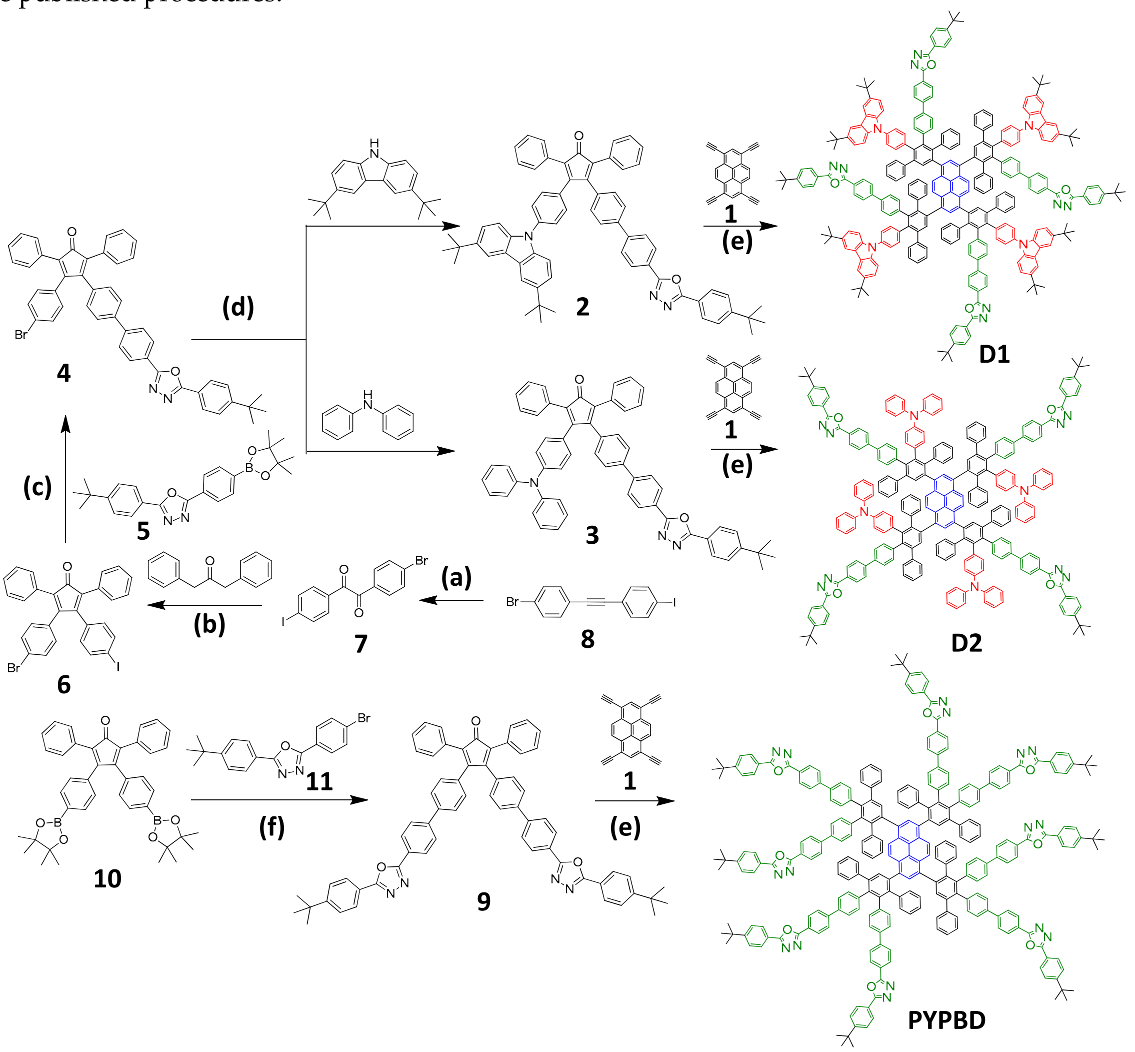

Scheme S1. Synthetic routes for the polyphenylene dendrimers and **CP**s *. * Reagents and conditions: (a) I_2, DMSO, 155 °C, 24 h, 89%; (b) 1,3-diphenylacetone, KOH, EtOH, 80 °C, 1 h, 76%; (c) $Pd(PPh_3)_4$, K_2CO_3, tetrabutylammonium bromide, toluene, H_2O, 48 h, 80 °C , 100%; (d) $Pd_2(dba)_3$, t-BuONa, $(t\text{-}Bu)_3P$, toluene, 24 h, 110 °C, 78% for **2**, 75 °C, 39% for **3**; (e) *O*-xylene, 130 °C, 24 h, 50% for **D1**, 45% for **D2**, 150 °C, 24 h, 26% for **PYPBD**; (f) $Pd(PPh_3)_4$, K_2CO_3, tetrabutylammonium bromide, toluene, H_2O, 48 h, 110 °C, 87%.

1-(4-Iodophenyl)-2-(4-bromophenyl) diketone (**7**)

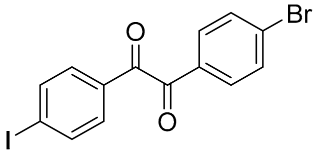

Compound **8** (4.0 g, 10.4 mmol), iodine (2.66 g, 10.44 mmol) and 130 ml of DMSO were added in a 250 mL flask. Then the mixture was degassed and added argon 3 times and stirred in 155 °C for 24 h. Then it was filtered and washed with DCM, THF and acetone. Eventually, 3.86 g light yellow solid was received after drying under reduced pressure (89.1%). ^{1}H-NMR (300 MHz, THF-d_8, 300 K) δ 7.95 (d, 8.7 Hz, 2H), 7.87 (d, 8.7 Hz, 2H), 7.73 (d, 8.7 Hz, 2H), 7.69 (d, 8.7Hz, 2H). ^{13}C-NMR (75 MHz, THF-d_8, 300 K) 192.99, 182.76, 139.20, 133.10, 132.04, 131.63, 130.68, 104.07. FD-Mass: calculated for $C_{14}H_8BrIO_2$: 413.9 (100%), found: 414.4 $[M]^+$.

3-(4-Bromophenyl)-4-(4-iodophenyl)-2,5-diphenylcylcopentadienone (**6**)

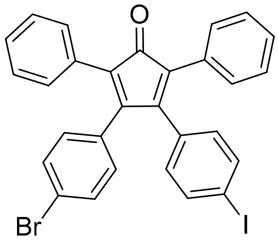

Compound **7** (3.0 g, 7.22 mmol), 1,3-diphenylacetone (1.51 g, 7.18 mmol) and 30 mL of ethanol were added in a 100 mL schlenk flask. Then the mixture was degassed and added argon 3 times and heated to 85 °C to make the solids dissolved. Then, 0.2 g of KOH in 5 ml ethanol was added. The mixture was stirred for another 1 h, followed by cooling with ice bath. Then water was added to form precipitate which was filtered and washed with methanol. Eventually, 3.21 g pure-colored solid was received after drying under reduced pressure (76%). ^{1}H-NMR (300 MHz, CD_2Cl_2, 300 K) δ 7.55 (d, 2H), 7.35 (d, 2H), 7.30–7.26 (m, 6H), 7.21–7.18 (m, 4H), 6.82 (d, 2H), 6.68 (d, 2H). ^{13}C-NMR (75 MHz, CD_2Cl_2, 300 K) 153.41, 137.79, 132.82, 132.28, 131.84, 131.45, 131.40, 130.90, 130.51, 128.56, 128.20, 123.37, 95.25. FD-Mass: calculated for $C_{29}H_{18}BrIO$: 588.0 (100%), found: 588.4 $[M]^+$.

3-(4-Bromophenyl)-4-[4-(2-tertbutylphenyl-5-phenyl-1,3,4-oxadiazolyl)phenyl]-2,5-diphenylcylcopentadienone (**4**)

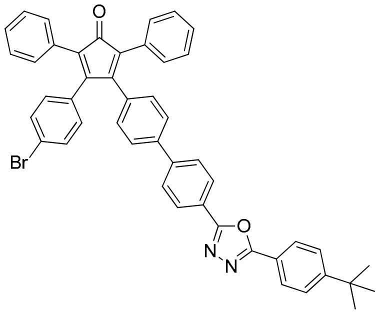

Compound **5** (1.00 g, 2.47 mmol), compound **6** (1.89 g, 3.22 mmol), tetrabutylammonium bromide (4 mg, 0.01 mmol), K_2CO_3 (0.68 g, 4.95 mmol), 60 mL of toluene and 20 mL of water were added in a 250 mL round bottom flask. Then the mixture was degassed and added argon for 3 times, followed by the addition of Pd $(PPh_3)_4$ (143 mg, 0.12 mmol). The mixture was then stirred at 80 °C for 48 h. Then it was extracted by DCM and purified by a silica gel flash column (first DCM, then ethyl acetate/DCM (5/95) as eluents). Eventually, 1.85 g of purple-colored solid was received after drying under reduced pressure (100%). ^{1}H-NMR (300 MHz, CD_2Cl_2， 300 K) δ 8.20 (d, J = 8.5 Hz, 2H), 8.08 (d, J = 8.6 Hz, 2H),

7.80 (d, *J* = 8.6 Hz, 2H), 7.58 (dd, *J* = 10.6, 8.5 Hz, 4H), 7.36 (d, *J* = 8.5 Hz, 2H), 7.28–7.23 (m, 10H), 7.07 (d, *J* = 8.4 Hz, 2H), 6.89 (d, *J* = 8.5 Hz, 2H), 1.38 (s, 9H). ^{13}C-NMR (75 MHz, CD_2Cl_2, 300 K) δ 165.16, 164.59, 155.94, 154.04, 153.58, 143.51, 140.23, 133.21, 132.53, 131.77, 131.52, 131.21, 131.04, 130.60, 130.55, 130.49, 129.40, 128.56, 128.53, 128.16, 128.10, 127.92, 127.71, 127.52, 127.09, 127.05, 126.58, 123.76, 123.30, 121.60, 35.44, 31.28. FD-Mass: calculated for $C_{47}H_{35}BrN_2O_2$: 738.2 (100%), found:738.1 $[M]^+$.

3-(4-(3,6-di-Tertbutylcarbazole)phenyl)-4-[4-(2-tertbutylphenyl-5-phenyl-1,3,4-oxadiazolyl)phenyl]-2,5-diphenylcylcopentadienone (**2**)

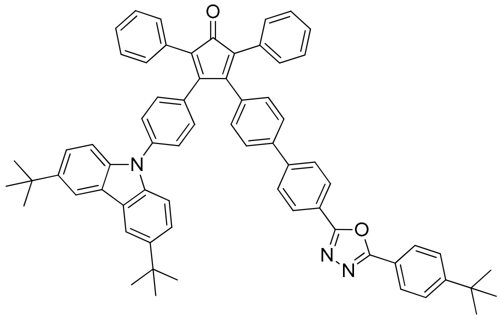

Compound **4** (1.52 g, 2.06 mmol) and 3,6-di-tertbutylcarbazole (0.59 g, 2.13 mmol) were placed in a 50 mL round bottom flask. It was then transferred to a glove box, in which sodium tertbutoxide (0.29 g, 3.04 mmol), tris(dibenzylideneacetone)dipalladium (46 mg, 0.05 mmol), tri(tertbutyl)phosphine (82 mg, 0.41 mmol) and 20 ml of dry toluene were added sequentially. Then the mixture was heated under reflux and stirred for 24 h. The resulting mixture was extracted with DCM and water, followed by a silica gel flash column, first using DCM then using ethyl acetate/DCM (2/98) as the eluent. It was further purified by recrystallization in ethanol. Eventually, 1.48 g yellowish brown solid was received after drying under reduced pressure (78%). ^{1}H-NMR (300 MHz, CD_2Cl_2, 300 K) δ 8.22 (d, *J* = 8.5 Hz, 2H), 8.14 (d, *J* = 1.6 Hz, 2H), 8.08 (d, *J* = 8.6 Hz, 2H), 7.83 (d, *J* = 8.5 Hz, 2H), 7.65–7.55 (m, 4H), 7.49–7.28 (m, 16H), 7.22 (d, *J* = 8.5 Hz, 2H), 7.17 (d, *J* = 8.4 Hz, 2H), 1.44 (s, 18H), 1.39 (s, 9H). ^{13}C-NMR (75 MHz, CD_2Cl_2, 300 K) δ 165.16, 164.59, 155.94, 154.39, 154.32, 143.73, 143.66, 140.26, 139.20, 138.74, 133.54, 132.06, 131.33, 130.64, 128.59, 128.56, 128.14, 128.10, 128.01, 127.72, 127.08, 127.05, 126.59, 126.28, 126.19, 126.12, 124.13, 123.93, 123.77, 121.61, 116.77, 109.60, 35.44, 35.05, 32.16, 31.30. FD-Mass: calculated for $C_{67}H_{59}N_3O_2$: 937.5, found: 937.8 $[M]^+$.

*3-(4-Triphenylamino)-4-[4-(2-tertbutylphenyl-5-phenyl-1,3,4-oxadiazolyl)phenyl]-2,5-diphenylcylcopentadienone (**3**)*

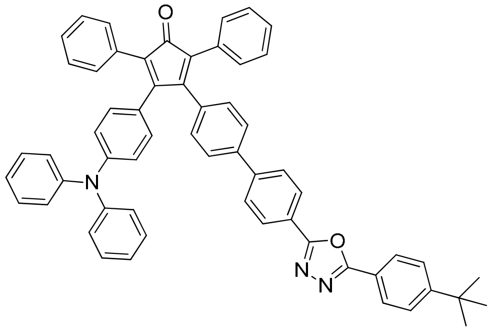

Compound **4** (1.00 g, 1.35 mmol) and diphenylamine (0.25 g, 1.49 mmol) were placed in a 50 mL round bottom flask. It was then transferred to a glove box, in which sodium tertbutoxide (0.19 g, 2.03 mmol), tris(dibenzylideneacetone)dipalladium (30 mg, 0.033 mmol), tri(tertbutyl)phosphine (55 mg, 0.27 mmol) and 15 mL of dry toluene were added sequentially. Then the mixture was heated at 75 °C and stirred for 24 h. The resulting mixture was extracted with DCM and water, followed by a silica gel flash column, first using DCM then using ethyl acetate/DCM (3/97) as the eluent. It was further purified by recrystallization in ethanol. Eventually, 0.44 g red-colored solid was received after drying under reduced pressure (39%). ^{1}H-NMR (300 MHz,

CD_2Cl_2, 300 K) δ 8.22 (d, *J* = 8.4 Hz, 2H), 8.14–8.04 (m, 2H), 7.85–7.75 (m, 2H), 7.59 (dd, *J* = 8.5, 7.1 Hz, 4H), 7.37–7.20 (m, 14H), 7.18–7.00 (m, 8H), 6.81 (s, 4H), 1.38 (s, 9H). ^{13}C-NMR (126 MHz, CD_2Cl_2, 298 K) δ 200.48, 165.16, 164.63, 155.94, 155.04, 154.04, 148.71, 147.50, 143.74, 140.02, 133.82, 131.90, 131.69, 131.51, 131.02, 130.69, 130.63, 130.58, 129.77, 129.67, 128.45, 128.42, 127.94, 127.92, 127.73, 127.71, 127.06, 126.84, 126.59, 126.46, 126.37, 125.83, 125.67, 125.52, 124.91, 124.88, 124.12, 123.70, 123.62, 123.46, 121.92, 121.62, 121.53, 54.06, 35.45, 31.29. FD-Mass: calculated for $C_{59}H_{45}N_3O_2$: 827.3, found: 827.9 $[M]^+$.

*3,4-Bis[4-(2-tertbutylphenyl-5-phenyl-1,3,4-oxadiazolyl)phenyl]-2,5-diphenylcylcopentadienone (**9**)*

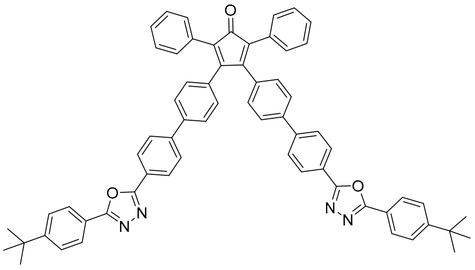

Compound **10** (0.809 g, 1.27 mmol), compound **11** (1.00 g, 2.80 mmol), potassium carbonate (0.879 g, 6.36 mmol), tetrabutylammonium bromide (82.5 mg, 0.254 mmol), 70 mL toluene, and 30 mL water were added into a 250 mL round bottom flask sequentially. The mixture was degassed and added argon 3 times, then $Pd(PPh_3)_4$ (73.5 mg, 0.0636 mmol) was added. The system was degased and added argon 3 times again and it was refluxed for 48 h. The resulting mixture was extracted with DCM/H_2O for 3 times and the organic layer was dried with $MgSO_4$, then filtered, condensed with rotary evaporation and run a silica gel flash column with ethyl acetate/DCM (10%–20%). Eventually, 1.03 g red brown solid was recovered after drying (86.6%). ^{1}H-NMR (300 MHz, CD_2Cl_2, 300K) δ 8.17(d, 4H), 8.05(d, 4H), 7.78(d, 4H), 7.59–7.54(m, 8H), 7.29(s, 10H), 7.12(d, 4H), 1.36(s, 18H). ^{13}C-NMR (75 MHz, CD_2Cl_2, 300K) δ 200.44, 165.12, 164.54, 155.95, 154.36, 143.59, 140.16, 133.47, 131.36, 130.63, 130.58, 128.52, 128.08, 127.89, 127.69, 127.02, 126.57, 126.42, 123.73, 121.60, 35.38, 31.26. FD-Mass: calculated for $C_{65}H_{52}N_4O_3$: 937.13, found: 937.0 $[M]^+$.

*Pyrene-Based, Peripheral-Carbazole-Oxadiazole Polyphenylene Dendrimer-G1 (**D1**)*

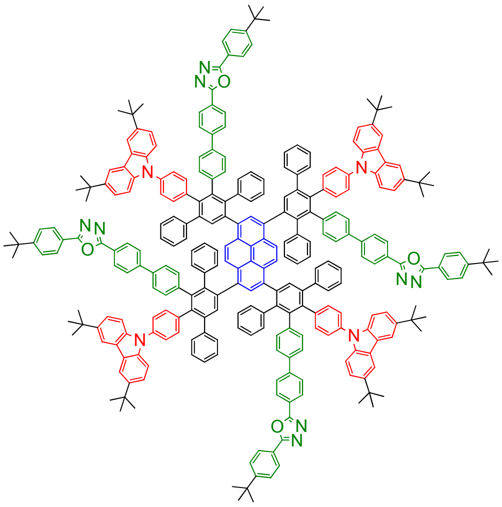

Compound **1** (15.0 mg, 0.05 mmol) and **2** (217.0 mg, 0.231 mmol) were placed in a 25 mL round bottom flask and dissolved in 8 mL anhydrous o-xylene. The mixture was stirred at 130 °C for 24 h. Then the cooled mixture was poured into hexane to precipitate and filtered. The obtained crude product was purified by a silica gel flash column (5/95, ethyl acetate/DCM). The dendrimer fractions were further purified by a GPC column (THF as the solvent). Eventually, 100 mg light yellow solid was received after drying under reduced pressure (50%). ^{1}H-NMR (300 MHz, CD_2Cl_2, 300 K) δ 8.20 (m, 8H), 8.09–8.06 (m, 20H), 7.82–6.61 (m, 110H), 1.39 (s, 36H), 1.36 (s, 72H). ^{13}C-NMR (126 MHz, CD_2Cl_2, 298 K) δ

165.03, 164.63, 155.87, 144.09, 144.04, 143.09, 143.04, 142.01, 141.92, 141.75, 141.84, 141.69, 141.41, 141.32, 141.06, 141.02, 140.91, 140.80, 140.75, 140.63, 140.56, 140.47, 140.43, 140.36, 140.32, 140.29, 140.25, 140.20, 140.14, 140.09, 140.02, 139.95, 139.86, 139.76, 139.71, 139.68, 139.63, 139.59, 137.28, 137.05, 136.92, 136.37, 136.16, 135.90, 135.58, 133.41, 133.35, 132.88, 131.89, 130.68, 130.59, 130.50, 128.21, 128.18, 127.87, 127.81, 127.64, 127.32, 127.00, 126.56, 126.14, 126.00, 125.78, 123.89, 123.40, 123.35, 123.29, 123.26, 121.60, 116.52, 109.33, 35.43, 34.94, 32.08, 31.27. MALDI-TOF: calculated for $C_{288}H_{246}N_{12}O_4$: 3937.9, found: 3939.5. HR-MALDI-TOF: calculated for $C_{288}H_{246}N_{12}O_4$: 3938.9516, found: 3939.0044 [M^+].

*Pyrene-Based, Peripheral-Triphenylamine-Oxadiazole Polyphenylene Dendrimer-G1 (**D2**)*

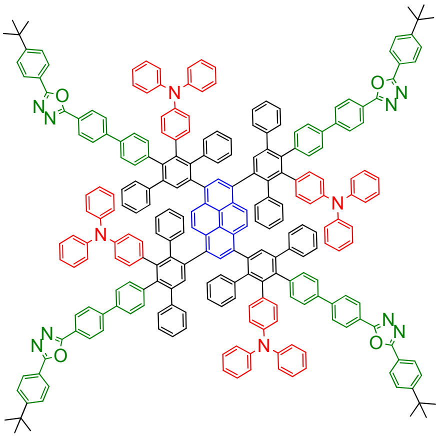

Compound **1** (15.0 mg, 0.05 mmol) and **3** (187.3 mg, 0.23 mmol) were placed in a 25 mL round bottom flask and dissolved in 7 mL anhydrous *O*-xylene. The mixture was stirred at 130 °C for 24 h. Then the cooled mixture was poured into hexane to precipitate and filtered. The obtained crude product was purified by a silica gel flash column (5/95, ethyl acetate/DCM). The dendrimer fractions were further purified by a GPC column (THF as the solvent). Eventually, 80 mg light yellow solid was received after drying under reduced pressure (45.4%). ^{1}H-NMR (300 MHz, CD_2Cl_2, 300 K) δ 8.18 (m, 8H), 8.08 (m, 8H), 8.00–6.52 (m, 138H), 1.38 (s, 36H). ^{13}C-NMR (126 MHz, CD_2Cl_2, 298 K) δ 165.06, 164.70, 155.85, 148.08, 145.83, 145.50, 144.16, 141.96, 141.35, 141.03, 140.73, 140.38, 139.95, 139.90, 137.17, 137.05, 137.02, 136.78, 136.33, 136.12, 136.04, 135.59, 132.97, 132.88, 131.92, 130.51, 129.39, 129.32, 128.10, 127.95, 127.69, 127.61, 127.01, 126.81, 126.56, 125.88, 125.54, 123.91, 123.71, 123.18, 123.12, 122.73, 122.58, 121.61. MALDI-TOF: calculated for $C_{256}H_{190}N_{12}O_4$: 3497.5, found: 3499.6. HR-ESI-MS: calculated for $C_{256}H_{190}N_{12}O_4$:3499.5256, found 1749.7679 [M^{2+}/2].

*Pyrene-Based, Peripheral-2-bis(p-phenyl)-5-(4-tertbutylphenyl)-1,3,4-oxadiazole polyphenylene dendrimer-G1 (**PYPBD**)*

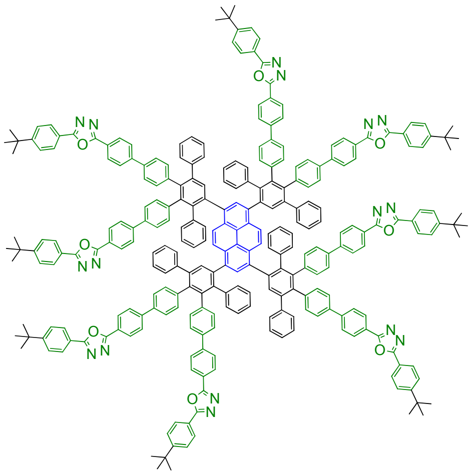

Compound **1** (20 mg, 0.067 mmol) and compound **9** (276 mg, 0.294 mmol) were stirred in 10 mL *O*-xylene at 150 °C for 24 h. The product was purified by silica gel flash column (EtAc/DCM, 1/4) and GPC column (THF). Eventually, 0.070 g of yellow-colored solid was received after drying under reduced pressure (26.5%). ^{1}H-NMR (250 MHz, CD_2Cl_2, 300 K) δ 8.20–7.81 (m, 38H), 7.75–7.49 (m, 36H), 7.43–6.42 (m, 72H), 1.35 (s, 72H). ^{13}C-NMR (176 MHz, CD_2Cl_2, 298 K) δ 165.01, 164.62, 155.82, 143.92, 141.94, 141.05, 140.76, 140.28, 137.23, 136.90, 136.33, 132.75, 130.46, 130.39, 128.29, 128.11, 128.07, 127.63, 127.56, 127.52, 126.97, 126.53, 126.01, 125.68, 123.16, 123.10, 121.57, 100.41, 35.40, 31.25. MALDI-TOF: calculated for $C_{280}H_{218}N_{16}O_8$: 3933.7, found: 3935.4. HR-ESI-MS: calculated for $C_{280}H_{218}N_{16}O_8$: 3936.7397, found: 1968.3635 [M^{2+}/2].

5. Molecular Structures of PYNPA, PYTPA and PYCAB

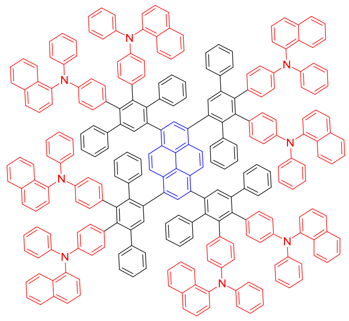

PYNPA

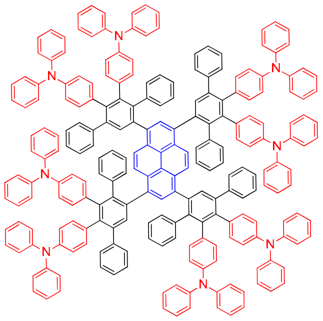

PYTPA

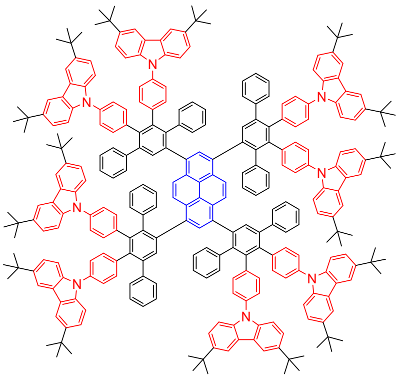

PYCAB

6. ^{1}H-NMR Spectra

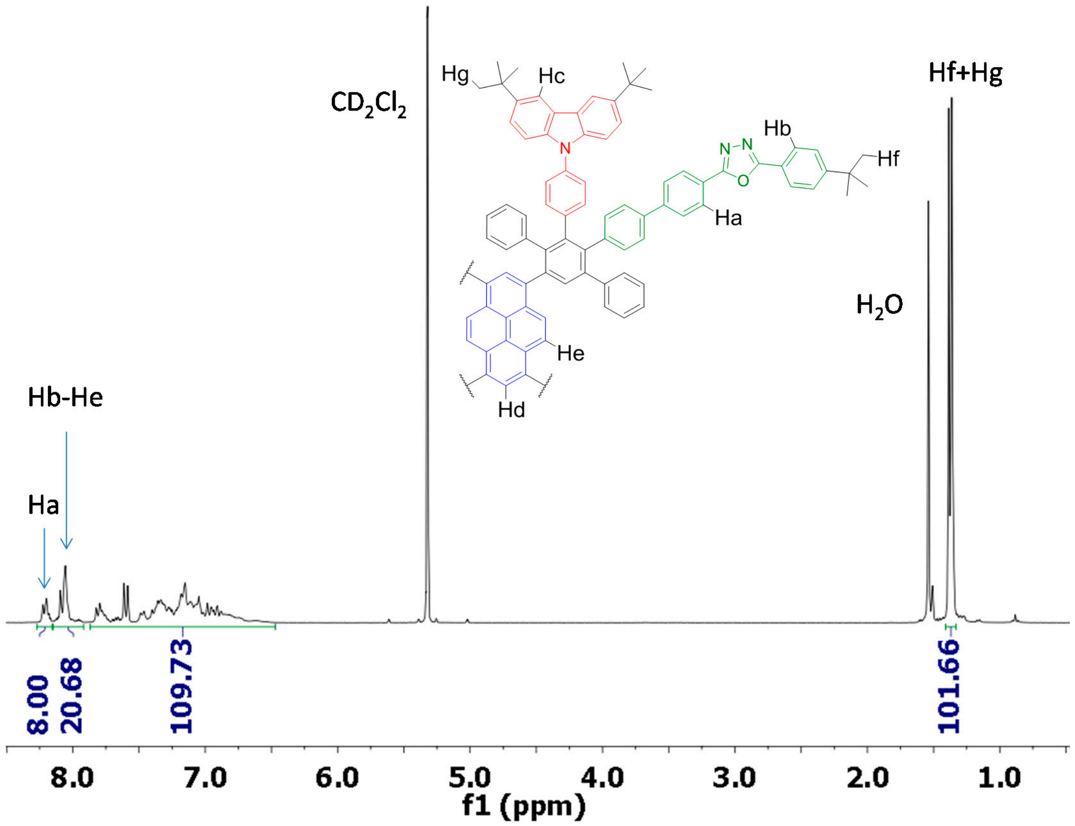

Figure S1. ^{1}H-NMR spectra of **D1** (300 MHz, CD_2Cl_2, 300 K).

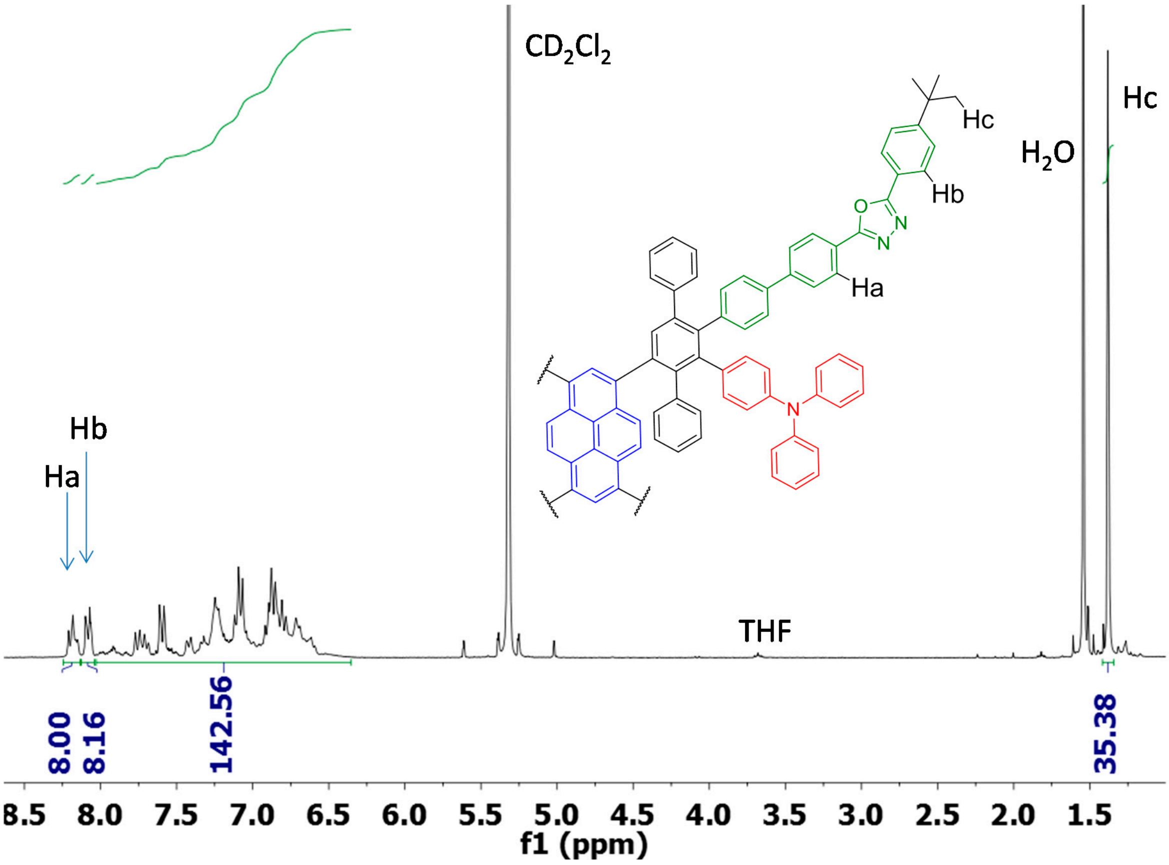

Figure S2. ^{1}H-NMR spectra of **D2** (300 MHz, CD_2Cl_2, 300 K).

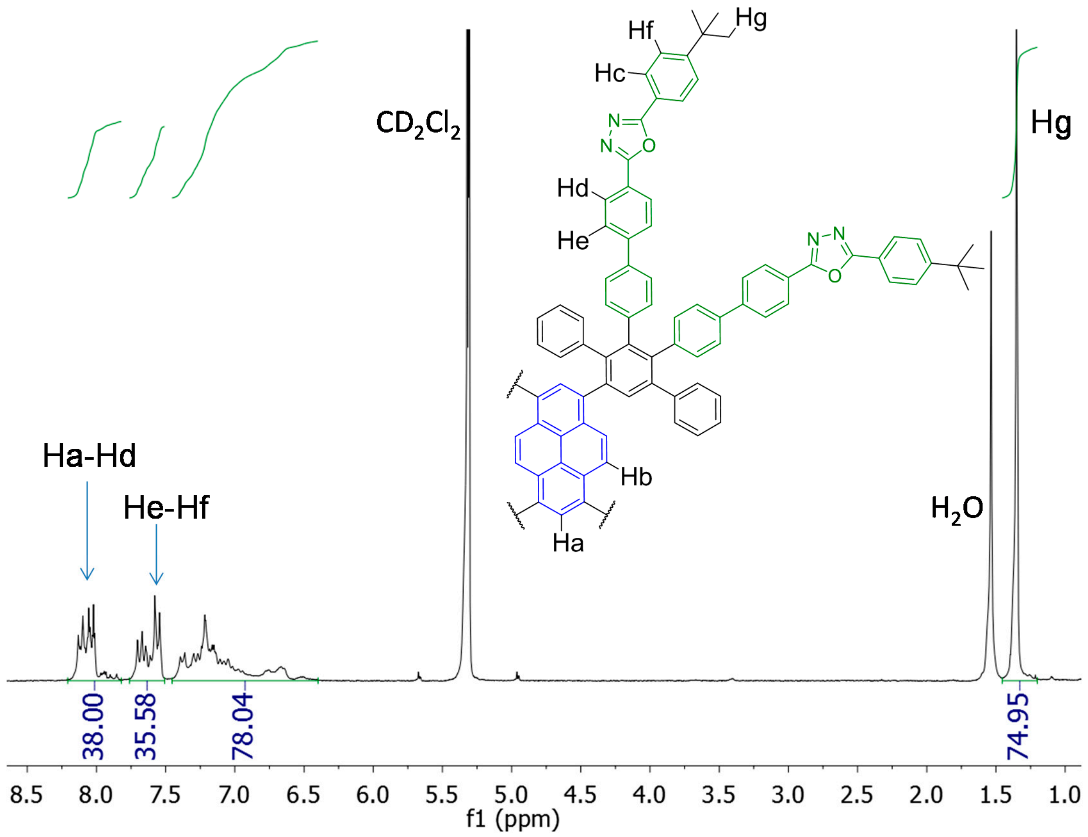

Figure S3. ^{1}H-NMR spectra of **PYPBD** (250 MHz, CD_2Cl_2, 300 K).

7. MALDI-TOF Mass and HRMS Spectra

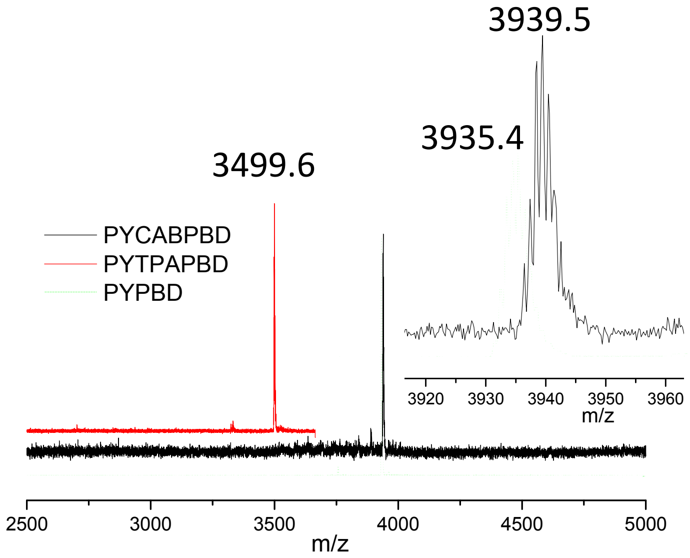

Figure S4. MALDI-TOF mass spectra of **D1**, **D2** and **PYPBD**.

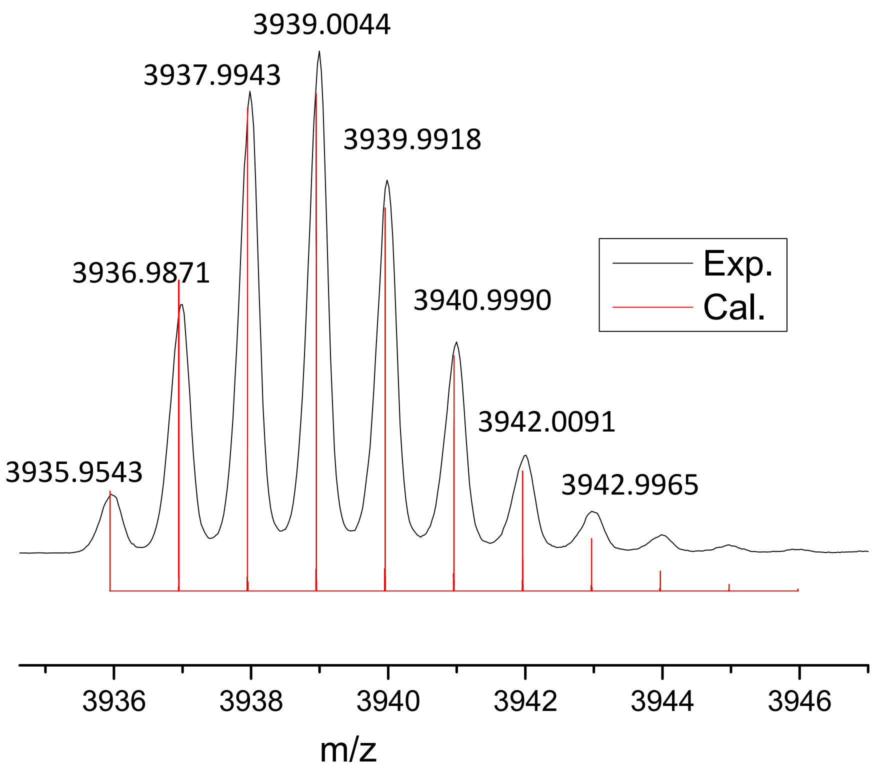

Figure S5. HR-MALDI-TOF mass spectra of **D1** ([M$^+$]).

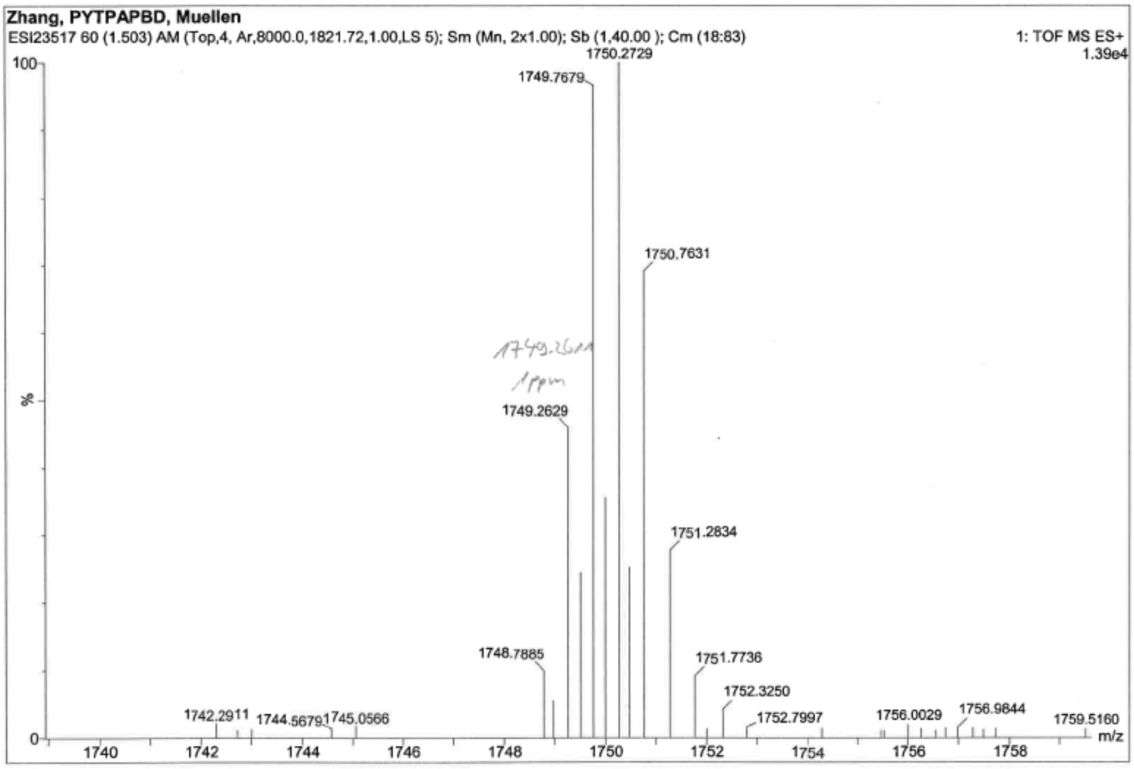

Figure S6: HR-ESI mass spectra of **D2** ([M^{2+}]).

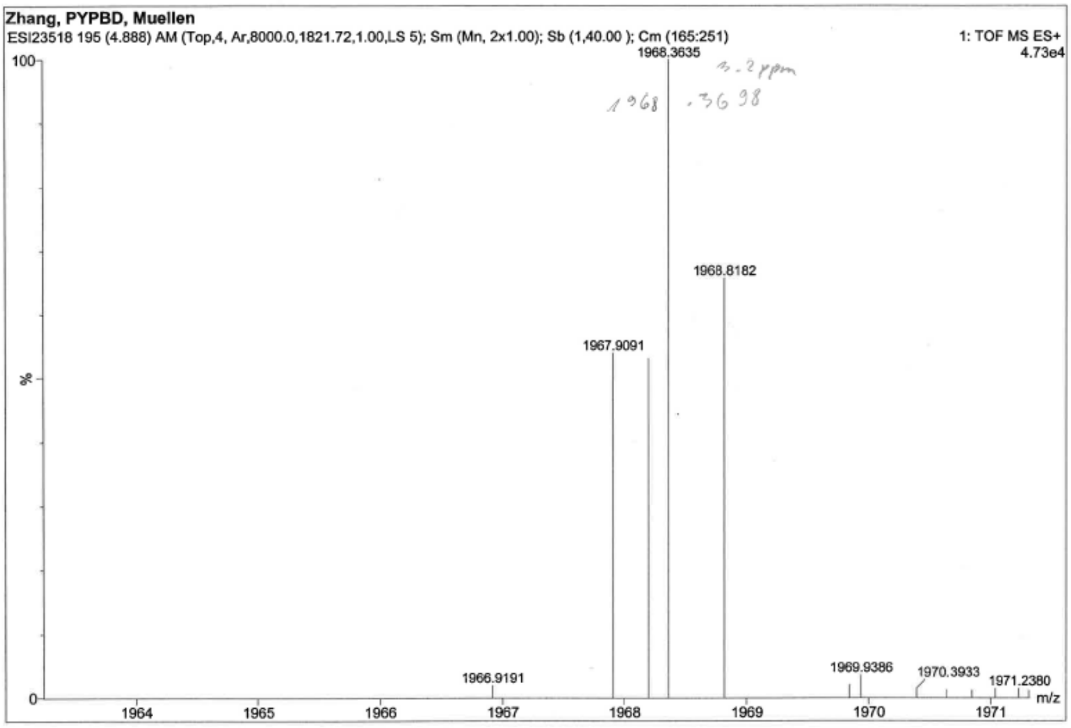

Figure S7. HR-ESI mass spectra of **PYPBD** ([M^{2+}]).

8. Crystal Structure of Compound 2

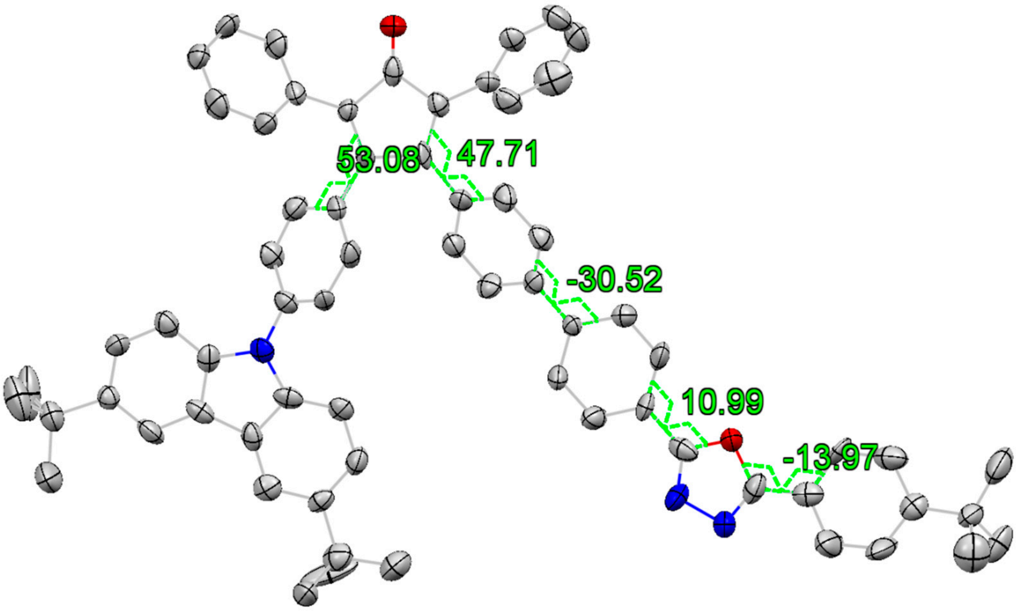

Figure S8. Crystal structure of compound **2** and the torsion angles.

Table S1. Single-Crystal X-ray Diffraction Parameters and Crystal Data for **2.**

**Formula**	**$C_{67}H_{59}N_3O_2$, $CHCl_3$**
molecular weight	1057.5 g·mol^{-1}
crystal system	triclinic
space group	$P\bar{1}$ (No. 2)
a (Å)	12.530(2)
b (Å)	13.193(3)
c (Å)	19.047(4)
α (deg)	98.185(5)
ß (deg)	103.738(5)
γ (deg)	107.851(5)
V (Å^3)	2177.6(3)
Z	2
T (K)	173(2)
Crystal description	brown needle
Absorption (mm^{-1})	0.21
crystal size (mm^3)	0.05 x 0.11 x 0.36
$2\theta_{min}$, $2\theta_{max}$ (deg)	4, 56
no. of reflns, (unique)	13509
no. of reflns ($I > 2\sigma(I)$)	1864
R1, wR2 (all data)	0.4249, 0.2375
R1 ($I > 2\sigma(I)$)	0.0848
GOF on F^2	0.701
Δ, e Å^{-3}	0.58, -0.50
CCDC	1489370

9. UV-vis Absorption and Photoluminescence Spectra

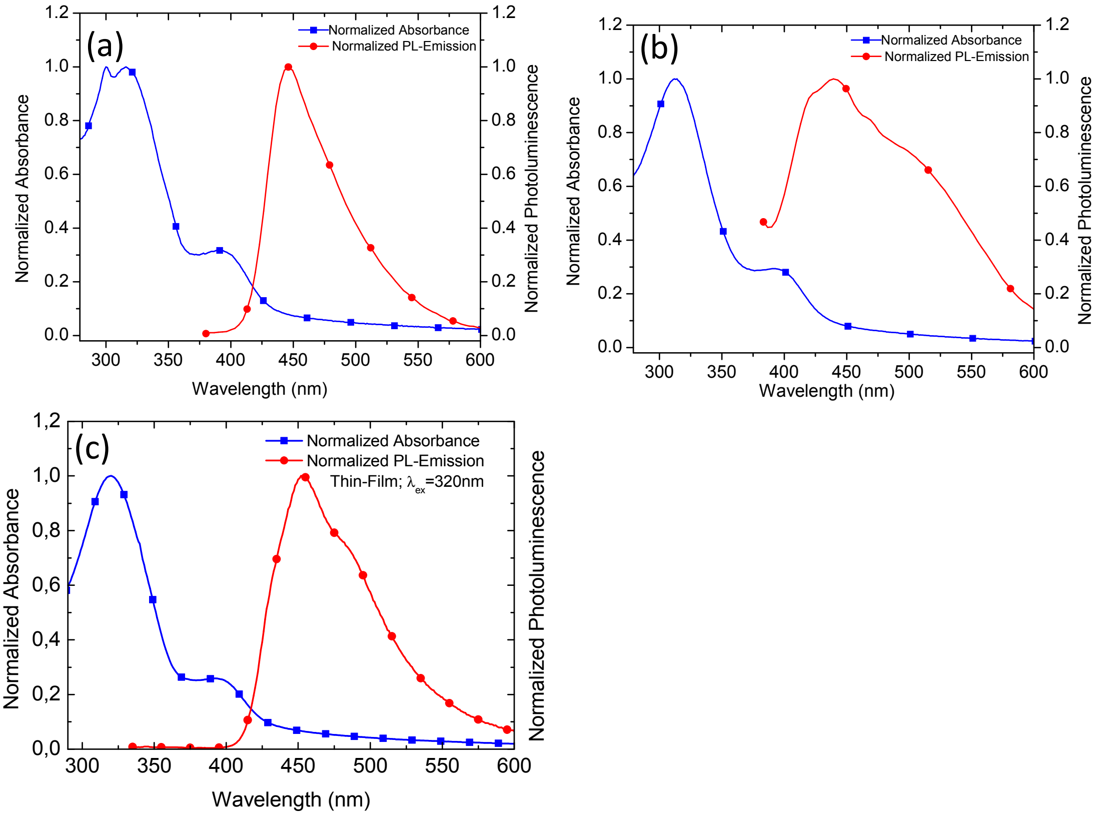

Figure S9. Thin-film absorption and Photoluminescence spectra (excited at 320 nm) of **D1** (**a**), **D2** (**b**) and **PYPBD** (**c**).

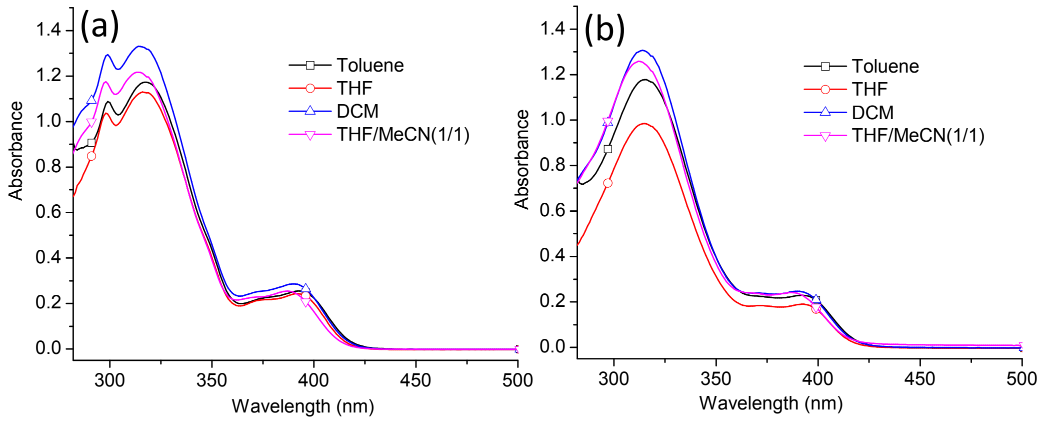

Figure S10. UV-vis absorption spectra of **D1** (a, 1.8 × 10^{-5} M) and **D2** (b, 2.0×10^{-5} M) in different solvents.

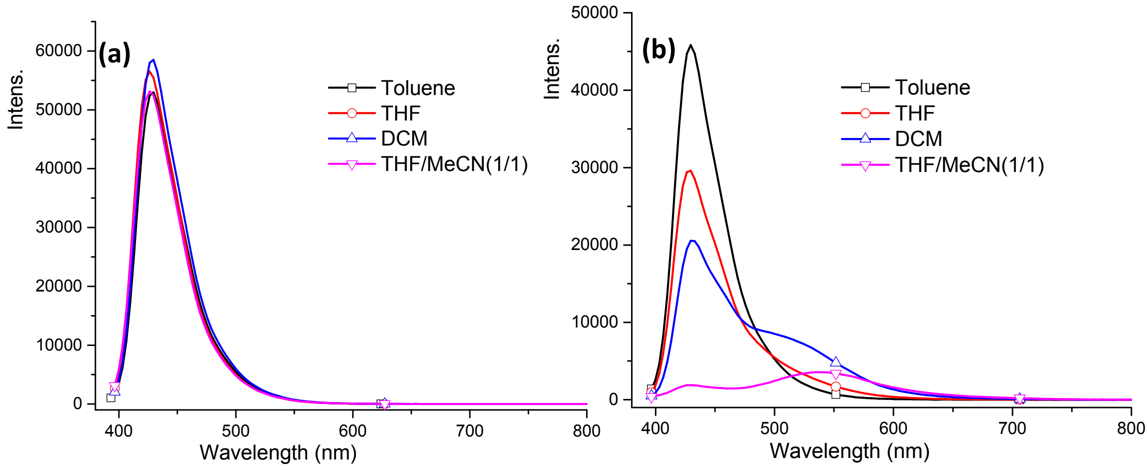

Figure S11: Photoluminescence spectra of **D1** (**a**) and **D2** (**b**) in different solvents (excited at 392 nm, 1.8 × 10^{-5} M (**a**), 2.0 × 10^{-5} M (**b**)).

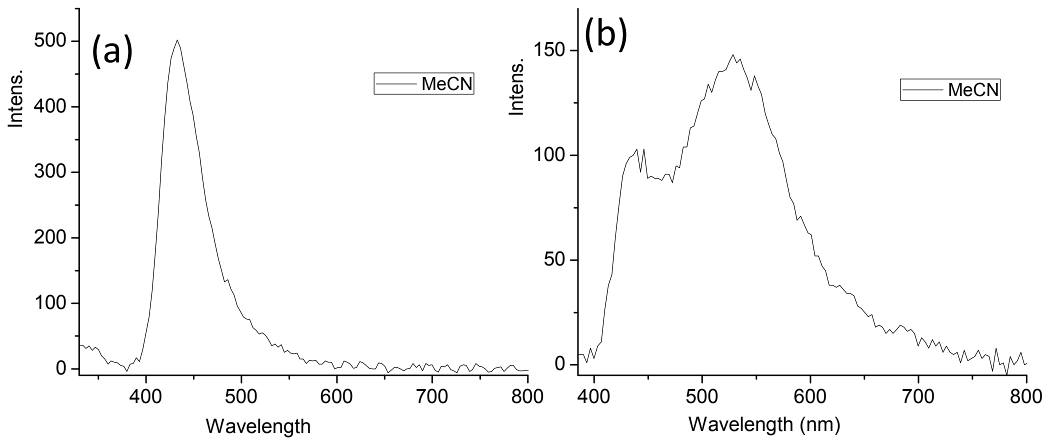

Figure S12. Photoluminescence spectra of **D1** (**a**) and **D2** (**b**) in acetonitrile. (note: the signals are very weak due to the very low solubility of these two dendrimers in acetonitrile, ex: 319 nm).

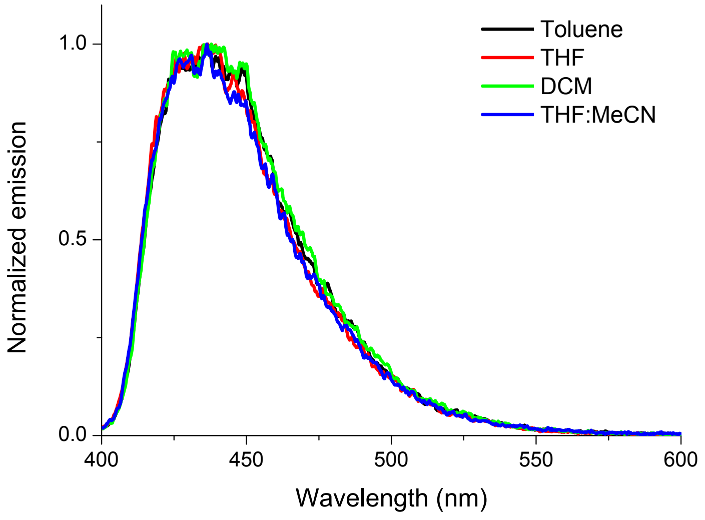

Figure S13. Emission spectra of **D1** in different solvents directly after excitation with 400 nm.

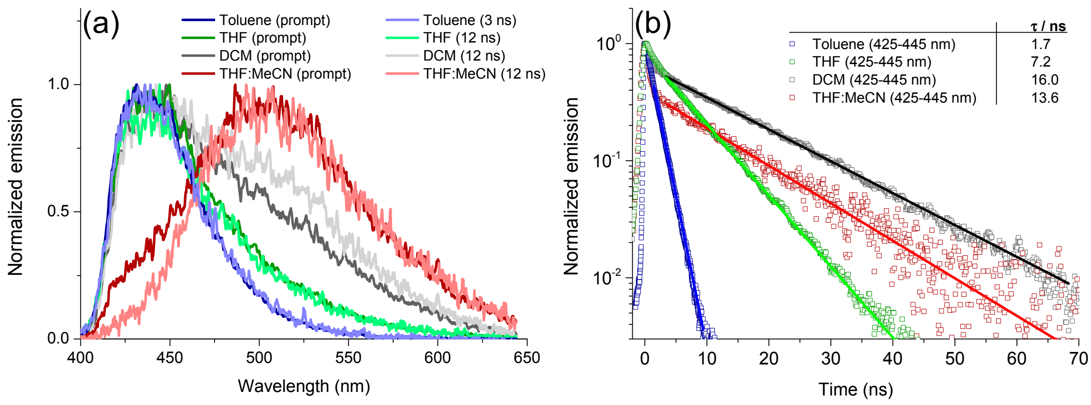

Figure S14. Emission spectra of **D2** in different solvent directly after excitation and at later delay (**a**); decay dynamics tracked from 425–445 nm in different solvent (open symbols) with monoexponential fits (solid lines) (**b**).

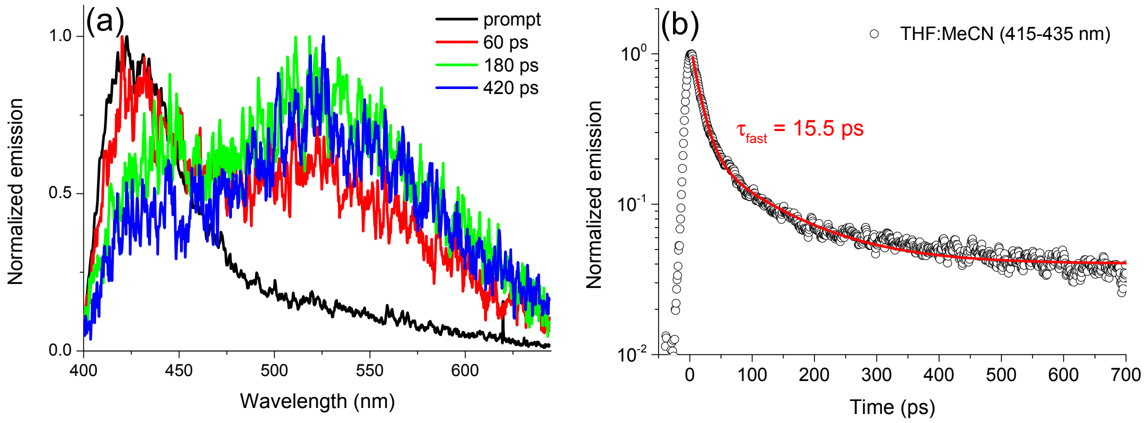

Figure S15. Emission spectra of **D2** in THF:MeCN at different delays after excitation with 400 nm on a picosecond timescale(**a**); decay dynamics tracked from 415–435 nm (black symbols) and a monoexponential fit including an offset (solid red line) yielding an inverse decay rate of 15.5 ps (**b**).

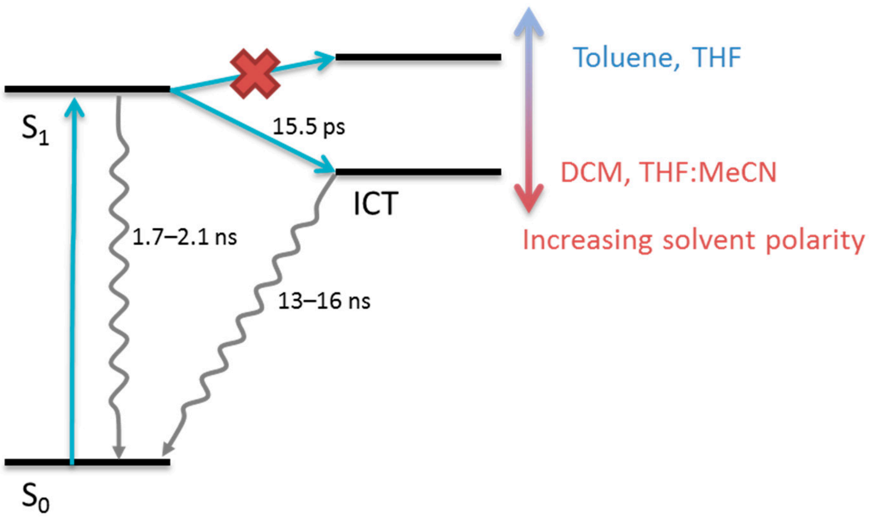

Scheme S2. Proposed energetic landscape for **D2** (note: the S_1 → ICT transition is only possible if the solvent polarity sufficiently stabilizes the ICT state).

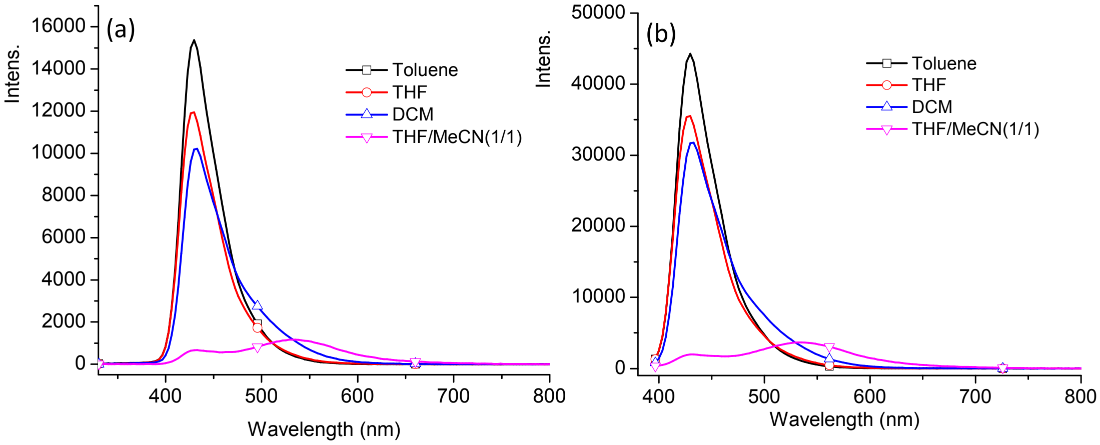

Figure S16. Photoluminescence spectra of **PYTPA** in different solvents, excited at 308 nm (**a**), and 392 nm (**b**) (3.9×10^{-6} M).

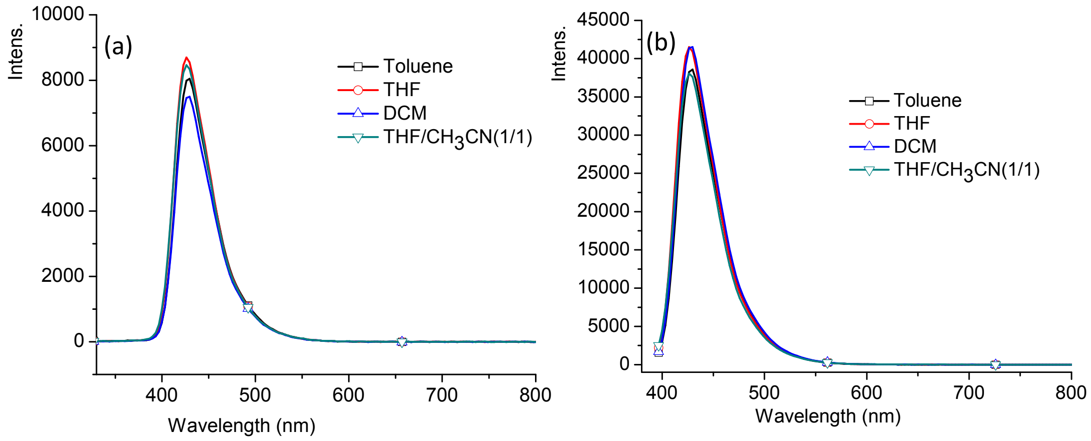

Figure S17. Photoluminescence spectra of **PYCAB** in different solvents, excited at 299 nm (**a**), and 392 nm (**b**) (4.0×10^{-6} M).

10. Structural Optimizations by DFT Method (B3LYP, 6-31g (d))

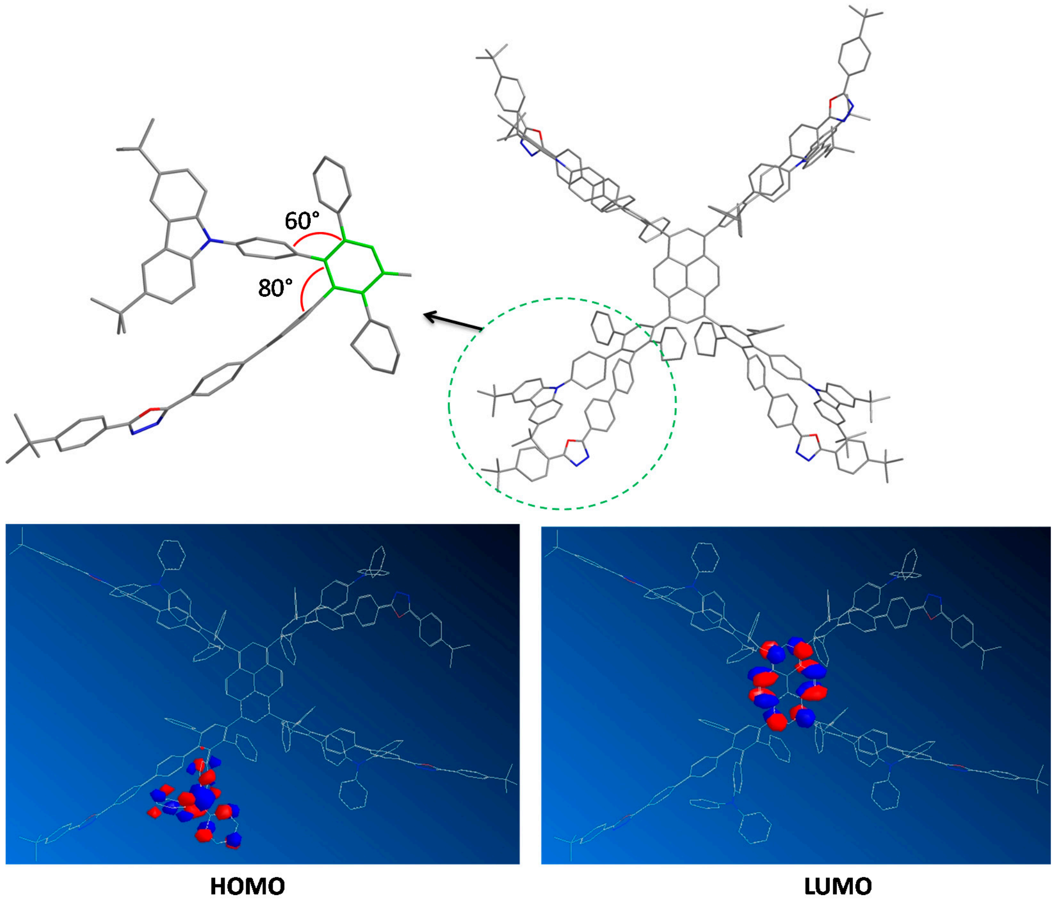

Figure S18. Structure optimization of **D1** (top) and **D2** (bottom) using DFT, B3LYP hybrid method and 6-31g(d) basis set.

11. Cyclic Voltammetry (CV) Curves

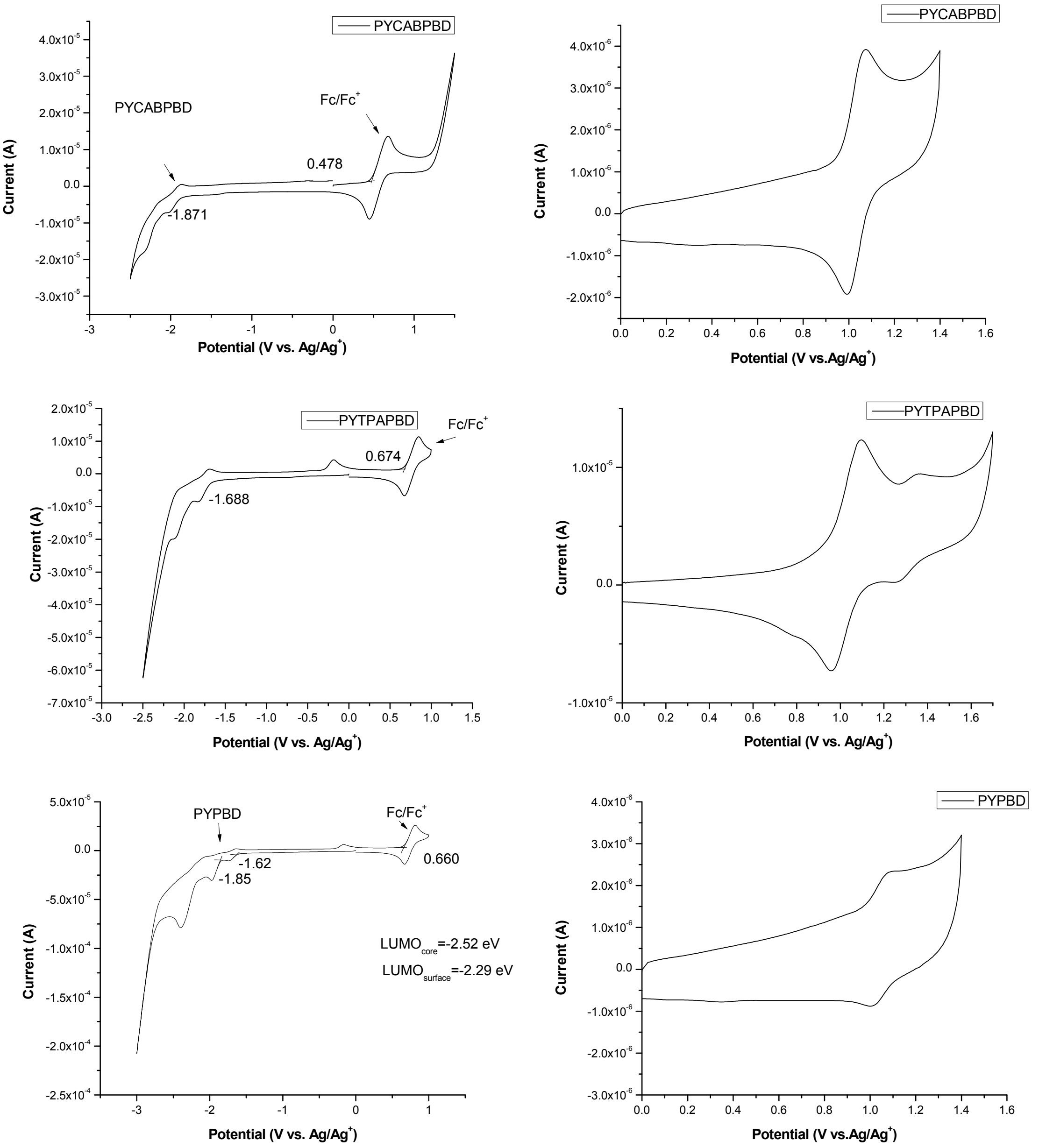

Figure S19: CV curves of **D1** (top), **D2** (middle) and **PYPBD** (bottom).

12. Electroluminescence Spectra

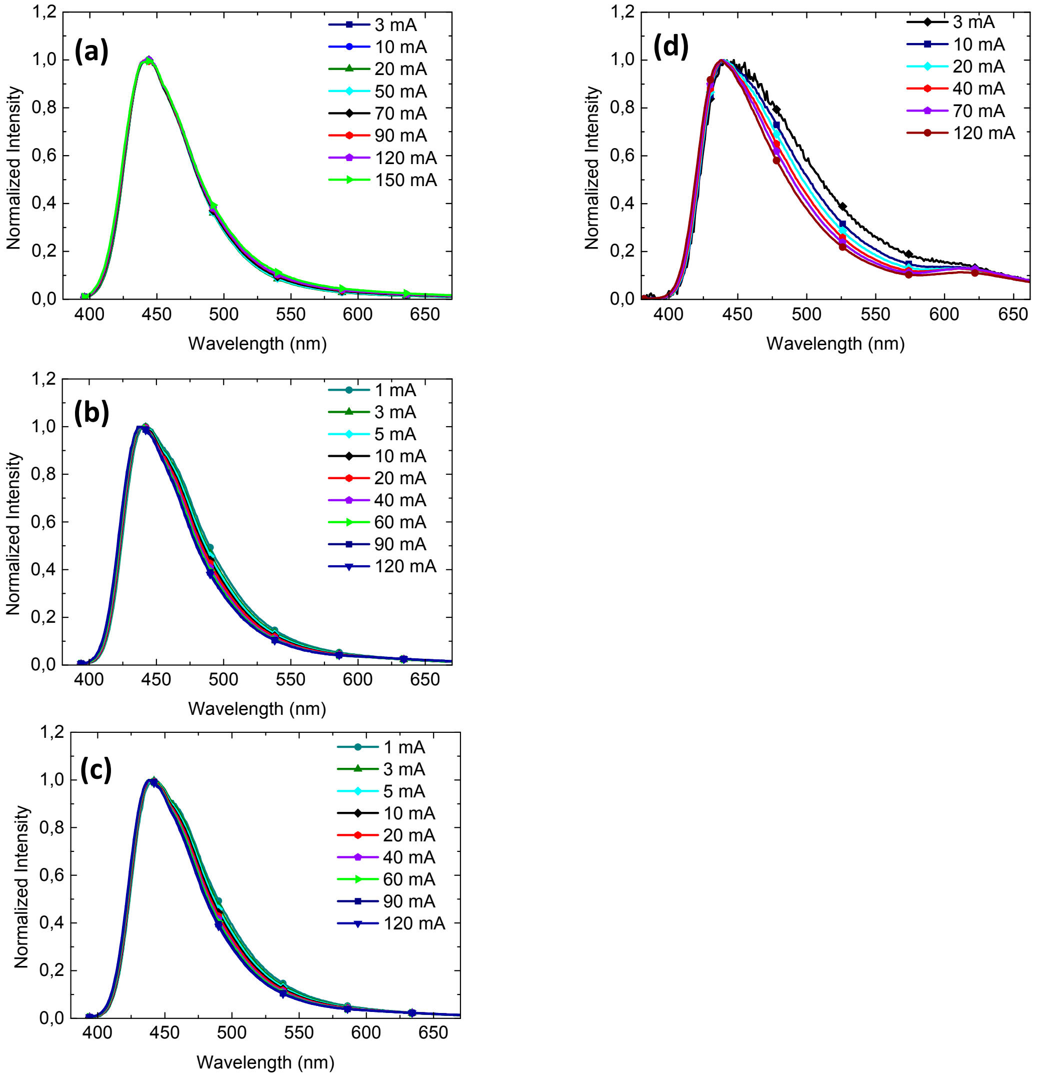

Figure S20. Electroluminescence spectra of **D1** in single-layer (**a**), two-layer (**b**) and three-layer (**c**) device and **D2** in two-layer device (**d**).

13. OLED performances of **PYNPA**

Table S2. OLED performances of **PYNPA**.

Dendrimer	V_{on} (V)	η (cd/A)	L_{max} (cd/m²)	CIE (x, y)
**PYNPA**	5.2	0.24	715	0.16, 0.20 [a]
	4	0.45	3726	0.16, 0.21 [b]

Device structure: [a] ITO/PEDOT:PSS/dendrimer/Ca/Al; [b] ITO/PEDOT:PSS/TFB/dendrimer/PEGPF/Cs_2CO_3/Ca.

14. References

1. Bernhardt, S.; Kastler, M.; Enkelmann, V.; Baumgarten, M.; Müllen, K. Pyrene as Chromophore and Electrophore: Encapsulation in a Rigid Polyphenylene Shell. *Chem. –Eur. J.* **2006**, *12*, 6117–6128.
2. Linton, K.E.; Fisher, A.L.; Pearson, C.; Fox, M.A.; Palsson, L.O.; Bryce, M.R.; Petty, M.C. Colour tuning of blue electroluminescence using bipolar carbazole-oxadiazole molecules in single-active-layer organic light emitting devices (OLEDs). *J. Mater. Chem.* **2012**, *22*, 11816–11825.
3. Orita, A.; Miyamoto, K.; Nakashima, M.; Ye, F.; Otera, J. Double Elimination Protocol for Convenient Synthesis of Dihalodiphenylacetylenes: Versatile Building Blocks for Tailor-Made Phenylene Ethynylenes. *Adv. Synth. Catal.* **2004**, *346*, 767–776.
4. Orita, A.; Taniguchi, H.; Otera, J. One-shot Double Elimination Process: A Practical and Concise Protocol for Diaryl Acetylenes. *Chem. -Asian J.* **2006**, *1*, 430-437.
5. Oesterling, I.; Müllen, K. Multichromophoric Polyphenylene Dendrimers: Toward Brilliant Light Emitters with and Increased Number of Fluorophores. *J. Am. Chem. Soc.* **2007**, *129*, 4595–4605.
6. Kamtekar, K.T.; Wang, C.S.; Bettington, S.; Batsanov, A.S.; Perepichka, I.F.; Bryce, M.R.; Ahn, J.H.; Rabinal, M.; Petty, M.C. New electroluminescent bipolar compounds for balanced charge-transport and tuneable colour in organic light emitting diodes: triphenylamine-oxadiazole-fluorene triad molecule. *J. Mater. Chem.* **2006**, *16*, 3823–3835.
